# QTL controlling fiber quality traits under salt stress in upland cotton (*Gossypium hirsutum* L.)

**DOI:** 10.1007/s00122-020-03721-x

**Published:** 2021-01-02

**Authors:** An-hui Guo, Ying Su, Yi Huang, Yu-mei Wang, Hu-shuai Nie, Nan Zhao, Jin-ping Hua

**Affiliations:** 1 Laboratory of Cotton Genetics; Genomics and Breeding/Key Laboratory of Crop Heterosis and Utilization of Ministry of Education/Beijing Key Laboratory of Crop Genetic Improvement, College of Agronomy and Biotechnology, China Agricultural University, No. 2, Yuanmingyuan West Rd, Haidian district, Beijing, 100193 China; 2 Oil Crops Research Institute, Chinese Academy of Agricultural Sciences, Wuhan, 430062 Hubei China; 3 Institute of Cash Crops, Hubei Academy of Agricultural Sciences, Wuhan, 430064 Hubei China

## Abstract

**Key message:**

QTL for fiber quality traits under salt stress discerned candidate genes controlling fatty acid metabolism.

**Abstract:**

Salinity stress seriously affects plant growth and limits agricultural productivity of crop plants. To dissect the genetic basis of response to salinity stress, a recombinant inbred line population was developed to compare fiber quality in upland cotton (*Gossypium hirsutum* L.) under salt stress and normal conditions. Based on three datasets of (1) salt stress, (2) normal growth, and (3) the difference value between salt stress and normal conditions, 51, 70, and 53 QTL were mapped, respectively. Three QTL for fiber length (FL) (*qFL-Chr1-1, qFL-Chr5-5, and qFL-Chr24-4)* were detected under both salt and normal conditions and explained 4.26%, 9.38%, and 3.87% of average phenotypic variation, respectively. Seven genes within intervals of two stable QTL (*qFL-Chr1-1* and *qFL-Chr5-5*) were highly expressed in lines with extreme long fiber. A total of 35 QTL clusters comprised of 107 QTL were located on 18 chromosomes and exhibited pleiotropic effects. Thereinto, two clusters were responsible for improving five fiber quality traits, and 6 influenced FL and fiber strength (FS). The QTL with positive effect for fiber length exhibited active effects on fatty acid synthesis and elongation, but the ones with negative effect played passive roles on fatty acid degradation under salt stress.

**Supplementary Information:**

The online version of this article (10.1007/s00122-020-03721-x) contains supplementary material, which is available to authorized users.

## Introduction

Soil salinity, as one of the major abiotic stresses, reduces global agricultural productivity due to the harmful effects on plant growth. Two main approaches can be used to produce salt-tolerant crops: (i) exploitation of natural genetic variations by direct selection or mapping quantitative trait loci (QTL) for subsequent marker-assisted selection (MAS); (ii) generation of transgenic plants to affect the degree of salt stress tolerance (Yamaguchi and Blumwald 2005). Salt stress is known to repress plant growth due to osmotic stress, which is then followed by ion toxicity. Drastic changes in ion and water homeostasis lead to molecular damage, growth arrest, and even death (Flowers 2004; Zhu 2001; Wang and Huang 2019; Richter et al. 2019). To overcome salt stress, complicated adjustment to metabolic networks with multipronged responses involves fast-acting, immediate physiological responses, and long-term reactions has been highlighted in plants under high salinity conditions (Guo et al. 2015; Zhang et al. 2011; Liu et al. 2019). Complex multi-component signaling pathways in plants can be triggered face salinity stress, such as plant hormones, plant transcription factor families (Su et al. 2017a), lipids (Gao et al. 2019), aquaporins (Wang et al. 2019; Pawłowicz and Masajada 2019), CIPK(Ma et al. 2019a, b), Snf (sucrose non-fermenting)-1-related protein kinases (SnRK2) (Su et al. 2017b; Shinozawa et al. 2019), and mitogen-activated protein kinase (MAPK), ROS signaling (Ismail and Horie 2017; Wang and Huang 2019) and stomatal signaling (Golldack et al. 2014).

Previous studies investigating salt tolerance was performed in QTL mapping at the germination stage or during vegetative growth, such as in cotton (Diouf et al. 2017; Sun et al. 2018, 2019), rice (Kumar et al. 2015), barley (Mano and Takeda 1997), tomato (Foolad and Chen 1999), and soybean (Lee et al. 2004). A major QTL for salt tolerance in soybean was discovered in 106 F_2_-derived lines from the cross of ‘S-100′ (salt tolerant line) × ‘Tokyo’ (salt sensitive line) with 41% of total genetic variation for salt tolerance in the field (Lee et al. 2004). The gene (*HKT1;4-A2*) was identified responsible to salt tolerance based on the QTL *Nax1*, which was mapped as a salt stress related QTL and linked to the microsatellite marker *gwm312* on chromosome 2AL in durum wheat (*Triticum monococcum*) in durum wheat (*Triticum monococcum*) (Huang et al. 2006; Tounsi et al. 2016). Therefore, more researches involving QTL exploration salt tolerance are imperative for further development in crop.

The damaging effects of salt accumulation in agricultural soils affect crop productivity due to soil salinity. Upland cotton, as an important crop for renewable natural fiber source of textiles, is a pioneer crop in saline areas, because it is tolerant with salinity threshold of 7.7 dS/m (77 mM NaCl), higher than soil saline with 4 dS/m (40 mM NaCl) (Chinnusamy et al. 2006; Shi et al. 2015). The release of genome data of upland cotton has greatly facilitated cotton research (Zhang et al. 2015; Hu et al. 2019a, b; Chen et al. 2020). Cultivated upland cotton adapted to various environments and experienced periodic salinity extremes (Wendel et al. 2010), originating from D sub-genome of response to abiotic stresses (Zhang et al. 2010). Eight SSR (simple sequence repeats) sites significantly associated with salt tolerance were found at the seedling stage through an association analysis in 134 cotton cultivars (Zhao et al. 2016). Comparative transcriptome analysis revealed that gas signaling process and ROS responding process enhanced abiotic stress in domesticated cotton and prolonged the duration of fiber elongation (Chaudhary et al. 2008, 2009; Park et al. 2012). One hundred and twenty-eight of the early differentially expressed proteins (DEPs) were identified from salt-treated cotton roots, 76 of which displayed increased abundance and 52 decreased under salt stress conditions in upland cotton (Li et al. 2015).

Obviously more exploration is needed in cotton, since no QTL under salt stress condition is included in 551 cotton QTL identified in Cottongen database resource (https://www.cottongen.org) (Ijaz et al. 2019). Some QTL clusters and hotspots containing 661 QTL were collected for traits response to salt stress in cotton, of which, 80 QTL were detected for salt tolerance just in greenhouse condition but no QTL in field condition (Abdelraheem et al. 2017). A total of 11 consistent QTL were detected for seven traits in seedling stage in an F_2:3_ population at 150 mM NaCl in a hydroponic environment (Oluoch et al. 2016). Recently, nine candidate intron length polymorphisms (ILPs) markers were verified using association mapping in a set of natural upland cotton accessions for salt stress in greenhouse condition (Cai et al. 2017). And the *C*4 gene encoding WRKY DNA-binding protein and the *C*9 gene encoding mitogen-activated protein kinase can significantly enhance cotton susceptibility to salt stress. The assessment of stress tolerance in the greenhouse often has little correlation with tolerance in the field (Yamaguchi and Blumwald 2005).

Cotton fiber is one of the most prevalent natural materials used in textile production. Fiber development consists of four stages (initiation, elongation, secondary cell wall biosynthesis, and maturation), which are defined and based on the number of days post-anthesis (DPA). For fiber development, the most active stage is the rapid elongation stage following the initiation stage and lasts up to 20 DPA (Kim et al*.* 2001). Studies have shown that the biosynthesis of very-long-chain fatty acids (VLCFAs, fatty acids > C18) plays important roles in fiber development as well as the precursors of sphingolipids, seed triacylglycerols, suberin, and cuticular waxes (Qin et al. 2007; Qin and Zhu 2011; Hu et al. 2019a, b). VLCFAs may activate ethylene synthesis in cotton fiber elongation (Shi et al. 2006). KCS (3-ketoacyl-CoA synthase) is the first limiting enzyme in the biosynthesis steps of VLCFA, which determines the substrate and tissue specificities of the reaction in plants (Qin et al. 2007). It is reported that 21 KCS genes were identified in the *Arabidopsis* genome with distinct tissue-specific, temporal-specific or spatial-specific expression patterns, reflecting their multiple roles in plant growth and development (Qin and Zhu 2011).

There is a lack of experimentation under natural salt stress conditions in the current literature. In the study, we evaluated field performance of five fiber quality traits under two conditions using a recombinant inbred lines (RIL) population derived from a cross of ‘Xinza 1′, an F_1_ hybrid upland cotton cultivar upland cotton. We performed QTL mapping of fiber quality traits under salt stress and normal growth conditions in three years in order to explore genetic basis of fiber development under salt stress in upland cotton. The findings of this research identify candidate genes associated with fiber elongation underlying the QTL and provide valuable insights for the improvement of cotton fiber quality.

## Materials and methods

### Plant materials

The RIL population was derived from a F_1_ hybrid ‘Xinza 1′ (GX1135 × GX100-2) by single seed descent method in upland cotton (Shang et al. 2015, 2016a), including 177 lines of F_15_–F_17_ generations. The control set was performed in two field trials, including GX1135, ‘Xinza 1′ F_1_, GX100-2, and a commercial hybrid ‘Ruiza 816′ used as a competitive check (Ma et al. 2017, 2019a, b).

### Field arrangement

Two field trials under salt stress and normal conditions were conducted in 2016, 2017, and 2018 at Quzhou Experimental Station of China Agricultural University, Handan City, Hebei Province (36°78′N, 114°92′E). Quzhou County is well known for the achievements in saline-alkali soil improvement since 1970′s.

Two independent field trials were arranged in neighboring fields following a randomized complete block design with two replications each in 2016, 2017 and 2018. A total of 362 plots with two rows (22 individual plants per row) were conducted, respectively. Two repeats of 177 RI Lines (F_15_–F_17_) were planted together with two control sets (GX1135, F_1_ ‘Xinza 1′, GX100-2, ‘Ruiza 816′). Each plot was spaced 80 cm apart, and rows within plots spaced 60 cm apart. Plot lengths were 2.4 m in 2016, and 3.0 m in 2017 and 2018. A 0.7 m pavement separated the field experiments.

For salt stress treatment, shallow saline groundwater with concentration of 5 g/L (85 mM) saline was used to irrigate the field twice in January and March before sowing. For control treatment, the regular irrigation with non-saline water was performed needed. Field management followed the local standard field practices.

### Soil sample collection and component detection

Soil samples were collected from the 0–20 cm and 20–40 cm depth after sowing or before harvest. To cover the experiment area, sampling points were chosen every 15 m from north to south in the experiment field. Three soil samples collected for each sample site were mixed into one sample for soil quality determination. Soil saturated paste extracts (1:2 by weight) were prepared to measure the electric conductivity (EC) and total content of water-soluble salt (*ρ*) (Rhoades 1996).

The measured sample properties of salinity from EC, and ρ for each sampling points are summarized in Table S1.

### Fiber sample preparation and evaluation

Twenty-five naturally opened bolls in the middle of plants were hand-harvested for each plot at crop maturity. A total of 367 and 363 fiber samples were prepared in 2016 on salt stress and normal conditions, respectively. In 2017, 362 and 358 fiber samples were collected from the two conditions. In 2018, a total of 362 fiber samples in each condition were collected. All samples were tested for five fiber quality traits with HVI 900 instrument (USTER_ HVISPECTRUM, SPINLAB, USA) at Cotton Fiber Quality Inspection and Test Center of Ministry of Agriculture (Anyang, China). The fiber quality traits obtained were as follows: 2.5% fiber span length (for short fiber length, unit: mm), fiber uniformity (%), fiber strength (cN/tex), fiber elongation (%), and fiber Micronaire (Shang et al. 2015; Ma et al. 2017).

### Dataset constitution and data conversion

Three datasets of (1) salt stress condition (E1), (2) normal condition (E2), and (3) the difference values between salt stress and normal conditions (*D*-value) were used in the present study. The original data of five fiber quality traits were obtained from the trials under E1 and E2, respectively. To ensure the *D*-value were positive, a constant (C = 10) was added to convert the data prior to statistical analysis.

### DNA extraction and marker detection

Genomic DNA was extracted from the parents and RIL plants using CTAB (Cetyltrimethylammonium bromide) method (Paterson et al. 1993). Genomic DNA of the RILs and two parents (GX1135 and GX100-2) was used to construct Illumina libraries with an insert size of 300–400 bp on the Illumina HiSeq platform. The clean reads were aligned to the *G. hirsutum* accession Texas Marker-1 (TM-1) reference genome using BWA software. The alignment files were converted into BAM files and then sorted using Samtools software (Li et al. 2009). The sorted reads in BAM files used in variant calling. SNP calling on a population scale was performed with the Genome Analysis Toolkit (GATK) (McKenna et al. 2010).

### Genetic linkage map construction

Linkage map analysis was conducted using Join Map 4.0. The adjacent markers from the same parent were recorded as one bin (Xie et al. 2010). The linkage map was constructed after the repetition was removed from the markers within the distance of 10 kb. 27,387 SNP (single nucleotide polymorphism), or InDel (insertion or deletion) markers were divided into 26 linkage groups by Logarithm of Odds (LOD) > 9, and 654 SSR markers involved in the original SSR genetic map (Shang et al. 2016b) were selected by LOD > 3. Finally, a total of 330 SSR markers were distributed in the new linkage map. Recombination frequencies were converted into map distances (cM) using the Kosambi mapping function (Kosambi 1944).

The Chi-square test was to determine if the observed genetic segregation ratios of alleles were consistent with expected segregation ratios. A region on the genetic map with at least six adjacent loci showing significant segregation distortion (*P* < 0.05) was defined as the segregation distortion region (SDR).

### Data analysis and QTL mapping

The experimental data were analyzed by the software SPSS (Version 20.0, SPSS, Chicago). QTL mapping and the genetic effect values at single-locus level were conducted by QTL Cartographer software (Version 2.5) using the composite interval mapping (CIM) method (Zeng 1994; Wang et al. 2007). We set parameter in the confidence interval of 95% with composite interval mapping (CIM) method for QTL mapping. The threshold of LOD values were estimated after 1000 permutations tests to declare a significant QTL with a significance level of *P* < 0.05, whereas QTL in another trial with LOD of at least 2.0 was considered as common QTL (Liang et al. 2013; Shang et al. 2015, 2016b). Common QTL were declared according to the position linked and if they shared one or two common markers (Shao et al. 2014).

### Candidate gene identification and annotation

The genes located in the confidence intervals of the important QTL were fetched from the Cottongen (https://www.cottongen.org) using their flanking marker positions in *G. hirsutum* TM-1 genome (Zhang et al. 2015) and considered as candidate genes. Gene ontology (GO) enrichment and KEGG pathway analysis were carried out for all candidate genes. The GO enrichment was performed using GO databases (https://archive.geneontology.org/latest-lite/). To further screen the possible candidate genes involved in fiber development, the gene expression pattern of candidate genes in different period of fiber development was analyzed using the cotton functional genome database (https://www.cottonfgd.org).

### RNA extraction and gene expression validation

Total RNA was isolated from 0, 5, 10, 15, and 20 DPA fiber in extremely long fiber length line (H), extremely short fiber length line (L), female (GX1135, F), and male (GX100-2, M). The concentration and purity of total RNA were determined using the Nano Drop spectrophotometry and agarose gel electrophoresis, respectively. RNA samples were stored at −80 °C freezer for future use.

To validate the potential function in fiber development, the expression patterns of candidate genes were verified with qRT-PCR using RNA of fiber in different development period of extremely lines in fiber length trait and parents of the population. Gene relative expression level was calculated with 2^−ΔΔCt^ method (Livak and Schmittgen 2001). Primers for the qRT-PCR analysis are listed in Table S3. Three independent replicates were performed for each sample. *GhUBQ7* gene was used as a reference gene.

## Results

### Phenotypic performance of fiber quality traits under salt stress and normal conditions

According to the grading standard of saline soil, the average EC of 0–20 cm soil samples collected during three years trial showed moderate salinization (323.11–648.22 μs·cm^−1^) in saline soil and mild salinization (350.08–455.33 μs cm^−1^) in normal soil, respectively (Table S1, Table S2) (Wang et al. 1993). The average EC of 20–40 cm was moderate salinization (656.78–943.92 μs cm^−1^) in saline soil and mild salinization (299.22–427.42 μs cm^−1^), respectively. It is indicated that there was a significant difference in salt concentration between the two areas after saline irrigation (Wang et al. 1993; Table S2).

For the datasets of E1, E2, and *D*-value, phenotypic performance of fiber quality traits is shown in Tables 1 and 2, including all lines from the RIL population, F_1_ hybrid ‘Xinza 1′, female parent GX1135, male parent GX100-2, and competitive check hybrid ‘Ruiza 816′ (Shang et al. 2015).

**Table 1:** Descriptive statistical analysis for fiber quality traits under salt stress and normal conditions

Traits	Year	Environment	Populations							Parents		F_1_	CK
			Mean	SD^ b^	CV% ^c^	Min	Max	Skewness	Kurtosis	GX1135	GX100-2	Xinza1	Ruiza 816
FL (mm)^ a^	2016	E1	30.28	1.17	3.85	26.75	33.90	− 0.13	0.31	30.23	29.70	30.43	31.00
	2016	E2	30.30	1.15	3.80	27.45	34.20	0.07	0.28	30.43	29.90	30.83	31.05
	2017	E1	30.52	1.15	3.78	26.70	33.05	− 0.28	0.05	30.30	30.95	30.40	29.35
	2017	E2	30.07	1.07	3.57	27.15	33.50	0.21	0.06	29.00	29.70	29.30	29.60
	2018	E1	29.39	1.08	3.66	26.50	32.10	0.08	− 0.27	28.05	28.70	28.45	30.25
	2018	E2	29.79	1.14	3.82	26.25	32.60	− 0.05	− 0.30	29.05	29.20	30.50	29.95
FU (%)	2016	E1	85.53	0.85	1.00	82.15	87.45	− 0.62	1.36	85.63	85.30	85.35	85.25
	2016	E2	85.77	0.73	0.85	83.90	87.40	− 0.31	− 0.22	86.30	85.55	86.53	85.20
	2017	E1	85.55	0.76	0.89	81.80	87.30	− 0.82	2.90	84.80	86.00	85.85	86.40
	2017	E2	84.76	0.86	1.01	82.90	87.10	0.01	− 0.57	85.30	85.30	86.35	86.65
	2018	E1	83.15	1.10	1.32	80.05	85.70	− 0.22	− 0.39	81.40	84.40	82.65	85.10
	2018	E2	84.36	0.89	1.06	82.10	86.80	− 0.12	0.01	82.55	84.25	85.45	85.35
FS (cN/tex)	2016	E1	28.58	1.22	4.25	25.80	31.65	− 0.07	− 0.24	30.45	27.60	28.60	30.43
	2016	E2	29.71	1.23	4.15	26.35	33.35	− 0.09	0.14	30.43	29.45	29.95	31.15
	2017	E1	28.98	1.64	5.68	25.45	34.40	0.22	0.16	28.95	29.00	29.00	29.75
	2017	E2	28.08	1.48	5.27	24.65	32.40	0.17	− 0.07	30.50	26.40	27.45	30.60
	2018	E1	31.49	1.83	5.80	26.55	36.30	0.11	− 0.02	31.35	30.10	29.95	33.90
	2018	E2	30.46	1.75	5.75	26.05	34.90	0.07	− 0.31	30.85	28.65	32.40	31.75
FE (%)	2016	E1	6.81	0.07	1.06	6.65	7.05	0.01	0.43	6.80	6.75	6.83	6.80
	2016	E2	6.78	0.08	1.17	6.50	6.95	− 0.23	0.37	6.78	6.78	6.88	6.83
	2017	E1	6.87	0.09	1.25	6.60	7.10	− 0.12	0.27	6.75	6.75	6.85	6.75
	2017	E2	6.80	0.07	1.10	6.60	7.05	0.13	0.46	6.75	6.75	6.85	6.75
	2018	E1	5.61	0.30	5.42	4.75	6.70	0.32	0.50	5.85	5.60	5.75	5.80
	2018	E2	5.70	0.33	5.72	4.65	6.45	− 0.33	0.17	5.75	5.50	6.00	5.80
FM	2016	E1	4.95	0.39	7.93	3.90	5.85	− 0.03	− 0.48	4.98	4.95	4.85	5.03
	2016	E2	4.83	0.38	7.93	4.05	5.75	0.23	− 0.73	4.78	4.40	5.10	5.10
	2017	E1	5.53	0.31	5.62	4.70	6.35	− 0.22	− 0.08	5.95	5.60	5.65	5.85
	2017	E2	5.58	0.28	5.02	4.60	6.25	− 0.38	0.57	5.70	5.55	5.95	5.75
	2018	E1	6.80	0.04	0.66	6.65	6.90	− 0.29	0.88	6.75	6.80	6.70	6.80
	2018	E2	6.80	0.05	0.80	6.70	6.95	− 0.01	− 0.13	6.75	6.80	6.90	6.75

E1, salt stress condition; E2, normal condition

^a^FL, fiber length/mm, FU, fiber uniformity ratio/%, FS, fiber strength(cN/tex), FE, fiber elongation/%, FM, Micronaire. Here in after same

^b^Mean values ± standard deviation values

^c^Coefficient of variation

**Table 2:** Descriptive statistical analysis for fiber quality traits on the difference value dataset between salt stress condition and normal condition

Traits	Year	Mean	SD	CV%	Min	Max	Skewness	Kurtosis	GX1135	GX100-2	Xinza1	Ruiza 816
FL (mm)	2016	9.99	0.95	9.53	4.95	12.70	− 0.57	4.26	9.80	9.80	9.60	9.95
	2017	10.44	0.98	9.39	7.85	12.90	− 0.10	− 0.41	11.30	11.25	11.10	9.75
	2018	9.60	0.84	8.79	7.55	12.35	0.00	0.02	9.00	9.50	7.95	10.30
FU (%)	2016	9.76	0.97	9.97	5.40	12.70	− 0.60	2.54	9.33	9.75	8.82	10.05
	2017	10.79	1.00	9.23	7.45	13.20	− 0.28	0.23	9.50	10.70	9.50	9.75
	2018	8.78	1.29	14.69	5.55	12.20	0.09	− 0.18	8.85	10.15	7.20	9.75
FS (cN/tex)	2016	8.87	1.21	13.64	5.00	12.65	0.03	0.96	10.02	8.15	8.65	9.28
	2017	10.90	1.52	13.96	6.65	15.70	0.14	− 0.11	8.45	12.60	11.55	9.15
	2018	11.01	1.66	15.07	6.00	15.30	− 0.34	− 0.03	10.50	11.45	7.55	12.15
FE (%)	2016	10.02	0.08	0.82	9.70	10.35	0.37	2.34	10.02	9.97	9.95	9.97
	2017	10.07	0.08	0.84	9.85	10.35	0.11	0.14	10.00	10.00	10.00	10.00
	2018	10.00	0.05	0.55	9.80	10.15	− 0.50	1.22	10.10	10.10	9.75	10.00
FM	2016	10.12	0.40	3.97	8.50	11.15	− 0.19	1.09	10.20	10.55	9.75	9.93
	2017	9.96	0.19	1.95	9.20	10.70	− 0.15	2.45	10.25	10.05	9.70	10.10
	2018	9.91	0.20	2.00	9.45	10.50	0.30	0.11	10.00	10.00	9.80	10.05

The performance of maternal parent GX1135 was often superior to that of male parent GX100-2 whether on E1 or E2. For GX1135 and GX100-2 with homozygous alleles, the trend of phenotypic values on E1 and E2 for fiber length (FL), fiber uniformity (FU) ratio and fiber strength (FS) across three years was not consistent. The phenotypic values on salt stress condition increased much for Micronaire (FM), but had no significant change for fiber elongation (FE) (Table 1). Moreover, mean value of F_1_ hybrid ‘Xinza 1′ for FM showed no incremental change. It revealed that responding mechanisms to salt stress may differ in the hybrid and its parents (Table 1).

In the RIL population, skewness and kurtosis values of five fiber quality traits ranged between −1.00 and 1.00 for both growing conditions, except that of FU under E1 in 2016 and 2017 (Table 1). Five fiber quality traits showed an abundance of variation in the in the RIL population either on E1 or E2. All fiber quality traits exhibited coefficient of variation less than 15%, indicating that the original data for these traits was reliable. Compared with E2, phenotypic values in the RIL population decreased for FL, FU and FS, but increased for FM and FE under E1 in 2016. In 2017, phenotypic values under E1 were increased for FL, FU, FS, and FE, but decreased for FM. In 2018, phenotypic values under E1 were decreased for FL, FU, and FE, however, increased for FS. These results indicate that the data obtained were credible.

### Correlation analysis between fiber quality traits

Table 3 presents correlation analysis on five fiber quality traits using datasets of mean phenotypic values on E1, E2, and *D*-value.

**Table 3:** Correlation analyses for five fiber quality traits under salt stress condition, normal condition, and by phenotypic differences

Environment	Trait	Year	FL	FU	FS	FE
E1	FU	2016	− 0.093			
	FS	2016	0.693**	− 0.132		
	FE	2016	0.560**	0.164*	0.510**	
	FM	2016	− 0.519**	0.349**	− 0.489**	− 0.143
	FU	2017	0.294**			
	FS	2017	0.768**	0.321**		
	FE	2017	0.559**	0.773**	0.578**	
	FM	2017	0.259**	0.756**	0.274**	0.840**
	FU	2018	0.196**			
	FS	2018	0.452**	− 0.017		
	FE	2018	0.579**	0.212**	0.540**	
	FM	2018	− 0.122	0.234**	− 0.453**	− 0.011
E2	FU	2016	0.380**			
	FS	2016	0.541**	0.273**		
	FE	2016	0.372**	0.212**	0.331**	
	FM	2016	− 0.390**	− 0.026	− 0.240**	0.126
	FU	2017	0.159*			
	FS	2017	0.528**	0.210**		
	FE	2017	0.382**	0.143	0.388**	
	FM	2017	− 0.509**	− 0.194**	− 0.289**	− 0.064
	FU	2018	0.232**			
	FS	2018	0.415**	0.178*		
	FE	2018	0.571**	0.171*	0.587**	
	FM	2018	− 0.169*	0.013	− 0.168*	0.103
*D*− value	FU	2016	0.379**			
	FS	2016	0.567**	0.366**		
	FE	2016	0.514**	0.454**	0.534**	
	FM	2016	− 0.516**	0.112	− 0.165*	− 0.001
	FU	2017	0.463**			
	FS	2017	0.544**	0.378**		
	FE	2017	0.524**	0.271**	0.588**	
	FM	2017	− 0.503**	− 0.091	− 0.194**	0.027
	FU	2018	0.296**			
	FS	2018	0.381**	0.178*		
	FE	2018	0.587**	0.100	0.552**	
	FM	2018	− 0.098	0.090	− 0.126	0.053

Critical value for correlation coefficients at probabilities of 0.05 and 0.01 are 0.145 and 0.190, respectively. E1, salt stress condition; E2, normal condition; *D*-value, the difference values between salt stress and normal conditions

^*^There was a significant correlation at 0.05 level (bilateral)

^**^There was a significant correlation at 0.01 level (bilateral)

Under E2, FL showed significant positive correlation with FS, FE, and FU, but was negatively correlated with FM during the three years of this trial. FS showed significant positive correlation with FE, and negative correlation with FM in three years. No significant correlation was observed between FU and FM. The results under E2 showed similar tendency and compared similarly to previous results (Liang et al. 2013; Shang et al. 2015). It revealed that the original data were reasonable for dissecting the genetic basis of fiber quality traits under both growing conditions.

Under E1, FL showed significant positive correlation with FS and FE, but showed negative correlation with FM in 2016, and significant positive correlation with FM in 2017. FU showed significant positively correlation with FE and FM during three years. FS showed significant positively correlation with FE each year, and significant positive correlated with FM in 2017, but was negatively with FM in 2016 and in 2018.

For FM, significant negative correlation existed with FL and FS under E2, but the correlative tendency under E1 was not consistent each year. FU showed significant positive correlation with FL and FS under E2 and showed significant positive correlation or no significant correlation under E1. It revealed that salt stress reduces the correlation between FL and FU, as well as FS and FU. FU showed significant positive correlation with FE and FM over three years, while the correlation between FU and FE, FU, and FM under E2 showed decreased, it indicated that salt stress enhanced the correlation dramatically. Correlations between FE and FM were not significant under E1 and E2. FE showed positive significant correlation with FS under both conditions. It indicated that salt stress had no effect on the correlations between FE and FS, FE, and FM.

### Construction of genetic map

Among the 174,351 markers, the low-coverage sequences of the RIL populations were filtered out, leaving 34,361 markers. After filtering SNPs according to the genotyping criteria, a total of 27,387 homozygous markers were identified between the two parents to generate bin markers for the RIL population. Finally, a total of 27,387 polymorphic SNP or Indel markers and 330 SSR markers were used for the construction of linkage map. Adjacent markers with the same genotype were merged as an identical bin. A high-density genetic linkage map was constructed with 2859 recombination bin markers. Distribution of markers and marker interval on chromosomes based on RIL linkage map is shown in Table 4. The genetic map covered 2133.53 cM of cotton genome with average interval of 0.785 cM. Among the 26 linkage groups, the average bin interval was 5.979 cM, and the range of interval on 26 chromosomes was 3.647–10.361 cM, and only one gap larger than 10 cM was observed in chromosome D05. The length of the linkage groups ranged from 34.181 cM (A09) to 149.966 cM (A05). The linkage map harboring these markers is shown in Table S4 and Fig. S1. On an average, one linkage group harbored about 110 bins that covered an average of 82.059 cM. The average bin interval was 0.758 cM with a range of 0.523 cM (A13) to 0.939 cM (D01). The highest number of markers (181) were present in the chromosome A05 with an average marker interval of 1.207 cM.

**Table 4:** Distribution of markers and marker interval on chromosomes based on RIL linkage map

Chr	Length(cM)	No. of markers	No. of bins	Marker interval (cM)	Bin interval (cM)	Max interval (cM)
A01	72.378	1646	109	0.044	0.670	6.410
A02	63.522	1115	98	0.057	0.655	3.979
A03	107.311	810	142	0.133	0.761	4.311
A04	76.875	1199	138	0.064	0.561	6.410
A05	149.966	960	181	0.156	0.833	5.690
A06	65.590	3618	104	0.018	0.637	3.979
A07	106.389	1021	129	0.104	0.831	6.050
A08	72.144	476	87	0.152	0.839	4.648
A09	34.181	583	45	0.059	0.777	4.991
A10	83.374	1170	107	0.071	0.787	5.365
A11	89.789	1783	129	0.050	0.701	5.690
A12	64.627	1094	104	0.059	0.627	3.647
A13	54.408	2366	105	0.023	0.523	3.647
D01	81.679	588	88	0.139	0.939	7.153
D02	103.223	1266	145	0.082	0.717	7.154
D03	87.538	1191	115	0.074	0.768	3.647
D04	69.669	326	76	0.214	0.929	6.781
D05	84.896	636	92	0.134	0.933	10.361
D06	85.986	787	124	0.109	0.699	5.340
D07	117.105	1227	153	0.096	0.770	6.049
D08	89.787	1078	120	0.083	0.755	6.050
D09	78.327	398	88	0.197	0.900	8.306
D10	65.318	394	78	0.166	0.848	8.305
D11	94.903	507	113	0.188	0.847	8.305
D12	87.626	558	113	0.157	0.782	6.410
D13	46.917	590	76	0.080	0.626	6.781
Whole	2133.528	27,387	2859	0.104	0.758	5.979

### Segregation distortion

Among the 2859 polymorphic loci, 336 (11.75%) showed segregation distortion (*P* < 0.05) with 213 (63.39%) favoring the GX1135 alleles and 123 (36.61%) favoring the GX100-2 alleles (Table S5). These distorted loci existed on 19 chromosomes and mapped unevenly on different chromosomes. More distorted loci were located on Dt subgenome than on At subgenome (142 versus 194). Among them, Chr. D01 and D04 accounted for 61.36% and 51.32% of distorted loci on corresponding chromosome, respectively (Table S5). There are 280 (83.33%) of the 336 distorted loci were clustered into 15 SDRs, with nine located on At subgenome and six on Dt subgenome. Chr. D11 was heavily concentrated with distorted loci, with 51 loci showing significant distortion toward GX100-2, forming a large SDR of 40.451 cM (Table S6). SDR12 which located on Chr. D04 showing significant distortion toward GX1135 and forming the largest interval of 40.561 cM (Table S6).

### QTL mapping for fiber quality

A total of 159 QTL controlling fiber quality traits were detected under E1 and E2, explaining 2.71–16.03% of total phenotypic variance (PV) (Table 5). Separately, 51, 70, and 53 QTL were detected in the RIL population under E1, E2, and *D*-value. Twelve QTL were detected in two or three years, of which seven, one, four, one, and one QTL were detected for FL, FU, FS, FE, and FM, respectively. A total of 10 QTL were identified in at least two datasets, three QTL were detected in E1 and E2 for FL, and one QTL was detected on E1 and *D*-value for FS. For FM, six QTL were detected on two datasets: three QTL on E1 and E2, two QTL on E2 and *D*-value, one QTL on E1 and *D*-value. However, no QTL were detected for FE and FU.

**Table 5:** Single locus QTL for fiber quality traits under normal condition, salt stress and mapping by the difference

Trait	QTL	Year	Flanking markers		Under E1^c^			Under E2			Mapping by *D*-value		
					LOD	Effect value	Var%^b^	LOD	Effect value	Var%	LOD	Effect value	Var%
FL	qFL-Chr1-1*^a^	2016	Bin28	Bin29	2.01	0.22	3.31	2.27	0.23	3.68			
		2018	Bin48	Bin49				2.90	– 0.28	5.83			
		2018	Bin51	Bin52	2.12	– 0.22	4.23						
	*qFL-Chr4-1*	2016	Bin400	Bin401	2.44	0.24	4.20						
	*qFL-Chr4-2*	2016	Bin421	Bin422	2.48	0.24	4.06						
	*qFL-Chr5-1*	2016	Bin490	Bin491				4.61	– 0.34	8.20			
		2017	Bin493	Bin494	6.49	– 0.40	12.11						
	*qFL-Chr5-2*	2016	Bin500	Bin501	2.79	– 0.26	4.87						
		2017	Bin502	Bin503	5.08	– 0.36	9.66						
	*qFL-Chr5-3*	2016	Bin512	Bin513				5.87	– 0.37	10.31			
		2017	Bin515	Bin516				6.23	– 0.36	11.06			
	*qFL-Chr5-4*	2017	Bin527	Bin528				6.90	– 0.38	12.14			
	qFL-Chr5-5*	2017	Bin648	Bin649				3.29	– 0.26	5.68			
		2016	Bin648	Bin649	5.91	– 0.39	10.80	6.73	– 0.40	11.65			
	*qFL-Chr6-1*	2016	Bin760	Bin761	3.84	0.31	6.82						
	*qFL-Chr7-1*	2016	Bin795	Bin796							2.49	0.23	5.57
	*qFL-Chr7-2*	2016	Bin837	Bin838							2.30	– 0.22	4.85
	*qFL-Chr7-3*	2017	Bin844	Bin845				3.34	– 0.28	6.35			
	*qFL-Chr7-4*	2017	Bin851	Bin852				2.30	– 0.22	4.02			
	*qFL-Chr9-1*	2018	Bin1024	Bin1025	2.35	– 0.24	4.70						
	*qFL-Chr11-1*	2017	Bin1168	Bin1169							2.23	– 0.21	4.48
	*qFL-Chr12-1*	2018	Bin1368	Bin1369							2.97	– 0.22	6.17
		2018	Bin1371	Bin1372				2.72	0.27	5.45			
	*qFL-Chr13-1*	2017	Bin1381	Bin1382	3.67	– 0.30	6.60						
	*qFL-Chr13-2*	2017	Bin1389	Bin1390	3.26	– 0.29	6.14						
	*qFL-Chr14-1*	2018	Bin1483	Bin1484	2.33	– 0.24	4.64						
	*qFL-Chr14-2*	2018	Bin1517	Bin1518				2.13	– 0.23	4.01			
	*qFL-Chr14-3*	2016	Bin1529	Bin1530							2.13	0.21	4.40
	*qFL-Chr15-1*	2017	Bin1599	Bin1600							2.47	– 0.23	5.15
	*qFL-Chr15-2*	2017	Bin1694	Bin1695				2.31	0.22	3.71			
		2018	Bin1707	Bin1708				2.02	0.23	4.00			
	*qFL-Chr16-1*	2016	Bin1760	Bin1761				2.13	– 0.21	3.26			
	*qFL-Chr16-2*	2017	Bin1784	Bin1785				2.10	– 0.20	3.38			
	*qFL-Chr18-1*	2017	Bin1933	Bin1934				2.07	– 0.21	3.33			
	*qFL-Chr19-1*	2017	Bin1996	Bin1997	2.38	– 0.24	4.21						
	*qFL-Chr21-1*	2017	Bin2335	Bin2336				2.84	0.24	4.87			
	*qFL-Chr21-2*	2016	Bin2338	Bin2339				3.19	0.28	5.65			
		2016	Bin2347	Bin2348				2.25	0.24	4.10			
	*qFL-Chr23-1*	2018	Bin2499	Bin2500							3.22	0.22	6.72
		2018	Bin2503	Bin2504	2.11	0.24	4.73						
	*qFL-Chr23-2*	2018	Bin2521	Bin2522	2.83	– 0.33	5.93						
	*qFL-Chr24-1*	2017	Bin2558	Bin2559	2.67	0.26	4.74						
	*qFL-Chr24-2*	2016	Bin2581	Bin2582				2.66	0.25	4.52			
		2016	Bin2587	Bin2588	2.61	0.26	4.67						
		2017	Bin2587	Bin2588				3.27	0.26	5.68			
	*qFL-Chr24-3*	2016	Bin2593	Bin2594				4.08	0.31	6.80			
	*qFL-Chr24-4*	2016	Bin2606	Bin2607	2.27	0.25	4.09	2.13	0.23	3.64			
	*qFL-Chr24-5*	2017	Bin2627	Bin2628							2.10	0.22	4.47
	*qFL-Chr25-1*	2017	Bin2674	Bin2675							2.07	– 0.21	4.35
	*qFL-Chr25-2*	2017	Bin2683	Bin2684							4.62	– 0.31	10.09
	*qFL-Chr25-3*	2017	Bin2690	Bin2691							4.22	– 0.29	8.61
	*qFL-Chr26-1*	2016	Bin2808	Bin2809	2.09	0.23	3.65						
FU	*qFU-Chr2-1*	2018	Bin110	Bin111							2.88	– 0.33	6.12
	*qFU-Chr4-1*	2017	Bin472	Bin473							3.10	– 0.26	6.48
	*qFU-Chr5-1*	2017	Bin492	Bin493				2.66	– 0.20	5.18			
	*qFU-Chr5-2*	2016	Bin554	Bin555							2.45	0.22	4.99
	*qFU-Chr6-1*	2016	Bin721	Bin722				2.09	– 0.15	4.21			
	*qFU-Chr7-1*	2017	Bin775	Bin776				2.04	– 0.17	3.79			
	*qFU-Chr7-2*	2017	Bin840	Bin841				2.20	– 0.18	4.39			
		2018	Bin857	Bin858				2.24	– 0.20	4.62			
	*qFU-Chr7-3*	2018	Bin867	Bin868				2.22	– 0.20	4.59			
	*qFU-Chr7-4*	2018	Bin881	Bin882				2.05	– 0.19	4.25			
	*qFU-Chr9-1*	2016	Bin991	Bin992				3.62	– 0.20	6.99			
	*qFU-Chr11-1*	2016	Bin1229	Bin1230				2.09	– 0.15	4.10			
		2016	Bin1239	Bin1240				3.49	– 0.19	6.72			
	*qFU-Chr13-1*	2016	Bin1374	Bin1375				4.29	0.22	8.24			
	*qFU-Chr13-2*	2017	Bin1449	Bin1450				2.55	0.20	5.03			
	*qFU-Chr13-3*	2018	Bin1467	Bin1468							2.15	0.30	4.54
	*qFU-Chr14-1*	2018	Bin1520	Bin1521				3.52	0.26	8.16			
	*qFU-Chr15-1*	2016	Bin1579	Bin1580				2.97	– 0.18	5.70			
	*qFU-Chr15-2*	2016	Bin1599	Bin1600							2.65	0.23	5.54
	*qFU-Chr15-3*	2017	Bin1620	Bin1621				2.25	0.19	4.84			
		2017	Bin1634	Bin1635				3.64	0.24	7.70			
	*qFU-Chr18-1*	2016	Bin1904	Bin1905	2.45	0.20	5.13						
	*qFU-Chr19-1*	2017	Bin2057	Bin2058	2.28	– 0.17	4.97						
	*qFU-Chr23-1*	2017	Bin2554	Bin2555							2.05	0.21	4.38
	*qFU-Chr24-1*	2016	Bin2558	Bin2559							3.37	– 0.30	6.93
	*qFU-Chr24-2*	2016	Bin2586	Bin2587							2.23	0.24	4.52
	*qFU-Chr24-3*	2017	Bin2630	Bin2631	2.74	0.19	5.78						
	*qFU-Chr25-1*	2016	Bin2681	Bin2682	2.20	0.18	4.54						
	*qFU-Chr25-2*	2018	Bin2763	Bin2764							2.70	– 0.31	5.72
		2018	Bin2774	Bin2775							2.76	– 0.32	5.85
FS	*qFS-Chr1-1*	2018	Bin3	Bin4				2.92	0.45	6.21			
	*qFS-Chr2-1*	2017	Bin139	Bin140							2.26	– 0.32	4.11
	*qFS-Chr5-1*	2016	Bin488	Bin489	3.60	– 0.31	6.50						
	*qFS-Chr5-2*	2016	Bin500	Bin501	2.31	– 0.25	4.24						
		2016	Bin502	Bin503				2.31	– 0.25	4.04			
	*qFS-Chr5-3*	2017	Bin548	Bin549	2.22	– 0.33	3.90						
	*qFS-Chr5-4*	2017	Bin629	Bin630				2.43	– 0.34	4.90			
	*qFS-Chr5-5*	2016	Bin638	Bin639				2.98	– 0.29	5.61			
	*qFS-Chr5-6*	2017	Bin640	Bin641	3.98	– 0.45	7.06						
	*qFS-Chr5-7*	2017	Bin653	Bin654	5.75	– 0.54	9.98						
		2016	Bin654	Bin655				5.75	– 0.40	10.42			
		2016	Bin655	Bin656	2.28	– 0.25	4.06						
	*qFS-Chr7-1*	2017	Bin789	Bin790				2.36	– 0.31	4.35			
	*qFS-Chr7-2*	2017	Bin804	Bin805							2.98	0.37	5.82
	*qFS-Chr7-3*	2018	Bin868	Bin869				2.27	– 0.41	5.11			
	*qFS-Chr7-4*	2017	Bin880	Bin881	2.10	– 0.33	3.74						
	*qFS-Chr8-1*	2018	Bin912	Bin913							2.27	– 0.37	4.85
	*qFS-Chr8-2*	2017	Bin975	Bin976	3.44	– 0.42	6.14						
		2017	Bin980	Bin981	2.75	– 0.38	5.00						
	*qFS-Chr11-1*	2017	Bin1144	Bin1145							4.05	– 0.44	7.95
	*qFS-Chr14-1*	2016	Bin1501	Bin1502				3.08	0.30	5.42			
	*qFS-Chr15-1*	2017	Bin1659	Bin1660				2.14	0.29	3.73			
	*qFS-Chr17-1*	2017	Bin1861	Bin1862							2.95	– 0.44	5.75
	*qFS-Chr17-2*	2016	Bin1870	Bin1871							2.06	0.29	4.36
	*qFS-Chr18-1*	2017	Bin1909	Bin1910							3.33	0.39	6.51
	*qFS-Chr19-1*	2017	Bin2066	Bin2067							2.75	0.36	5.35
	*qFS-Chr22-1*	2018	Bin2411	Bin2412							2.72	0.52	6.64
	*qFS-Chr24-1*	2017	Bin2578	Bin2579	6.25	0.56	11.19						
		2017	Bin2579	Bin2580				2.90	0.35	5.39			
		2016	Bin2581	Bin2582				2.60	0.27	4.66			
		2016	Bin2584	Bin2585	4.06	0.35	7.94						
	*qFS-Chr24-2*	2016	Bin2593	Bin2594	6.83	0.44	12.90						
		2017	Bin2592	Bin2593	7.19	0.60	12.73						
		2017	Bin2596	Bin2597				2.74	0.34	5.10			
	*qFS-Chr24-3*	2017	Bin2601	Bin2602	4.78	0.51	8.72						
	*qFS-Chr25-1*	2016	Bin2707	Bin2708	2.03	– 0.24	3.66						
		2016	Bin2712	Bin2713	2.67	– 0.28	4.78						
	*qFS-Chr25-2*	2016	Bin2769	Bin2770	3.21	0.30	5.79				2.17	0.27	4.95
FE	*qFE-Chr2-1*	2017	Bin128	Bin129							2.95	– 0.02	6.44
	*qFE-Chr5-1*	2016	Bin493	Bin494				5.49	– 0.03	11.13			
	*qFE-Chr5-2*	2016	Bin521	Bin522				3.26	0.02	6.42			
	*qFE-Chr5-3*	2016	Bin530	Bin531				2.13	0.02	4.36			
	*qFE-Chr7-1*	2018	Bin872	Bin873				3.33	– 0.01	6.80			
		2018	Bin879	Bin880							5.28	0.02	11.19
	*qFE-Chr9-1*	2017	Bin1010	Bin1011				3.18	– 0.02	6.10			
	*qFE-Chr10-1*	2017	Bin1123	Bin1124	3.04	0.02	5.98						
	*qFE-Chr10-2*	2017	Bin1132	Bin1133	3.72	0.02	7.44						
	*qFE-Chr11-1*	2018	Bin1192	Bin1193				3.16	– 0.01	6.43			
	*qFE-Chr12-1*	2016	Bin1364	Bin1365	2.08	0.02	4.65						
	*qFE-Chr14-1*	2018	Bin1494	Bin1495	3.65	– 0.01	7.66						
	*qFE-Chr14-2*	2018	Bin1508	Bin1509	4.60	– 0.01	9.54						
		2018	Bin1516	Bin1517	3.17	– 0.01	6.68						
	*qFE-Chr14-3*	2018	Bin1553	Bin1554				2.25	0.01	4.60			
	*qFE-Chr15-1*	2016	Bin1589	Bin1590	2.04	0.02	4.52						
		2016	Bin1596	Bin1597							2.80	0.02	5.68
		2016	Bin1599	Bin1600	2.08	0.02	4.22						
	*qFE-Chr16-1*	2016	Bin1718	Bin1719				2.94	– 0.02	5.94			
	*qFE-Chr17-1*	2017	Bin1835	Bin1836				2.17	0.02	4.35			
	*qFE-Chr18-1*	2018	Bin1971	Bin1972				3.04	– 0.01	6.19			
	*qFE-Chr19-1*	2018	Bin2089	Bin2090				2.42	– 0.01	4.87			
	*qFE-Chr19-2*	2018	Bin2099	Bin2100				2.37	– 0.01	4.78			
	*qFE-Chr20-1*	2017	Bin2185	Bin2186				3.76	– 0.02	7.13			
	*qFE-Chr20-2*	2018	Bin2204	Bin2204	2.05	0.01	3.90						
	*qFE-Chr20-3*	2016	Bin2248	Bin2249							4.64	0.03	10.43
		2016	Bin2254	Bin2255							3.62	0.02	7.41
		2017	Bin2267	Bin2268				2.07	0.01	3.63			
	*qFE-Chr21-1*	2018	Bin2279	Bin2280							4.21	– 0.02	8.83
	*qFE-Chr21-2*	2017	Bin2336	Bin2337				2.56	0.02	4.76			
	*qFE-Chr21-3*	2017	Bin2345	Bin2346	2.28	0.02	4.37						
	*qFE-Chr22-1*	2017	Bin2393	Bin2394							2.90	0.02	5.96
	*qFE-Chr22-2*	2017	Bin2412	Bin2413				2.55	0.02	4.44			
	*qFE-Chr24-1*	2018	Bin2584	Bin2585	3.70	0.01	7.59						
	*qFE-Chr25-1*	2017	Bin2695	Bin2696							2.90	– 0.02	5.95
FM	*qFM-Chr1-1*	2016	Bin96	Bin97				3.13	0.10	6.03			
	*qFM-Chr2-1*	2017	Bin128	Bin129				2.71	– 0.06	4.02			
	*qFM-Chr4-1*	2018	Bin360	Bin361				2.75	0.08	5.74			
		2018	Bin366	Bin367				2.18	0.07	4.58			
	*qFM-Chr4-2*	2017	Bin484	Bin485				3.99	– 0.07	5.72			
	*qFM-Chr5-1*	2017	Bin488	Bin489				2.21	0.05	2.91			
	*qFM-Chr5-2*	2017	Bin641	Bin642				6.00	0.09	9.37			
		2017	Bin645	Bin646	4.85	0.09	7.98	8.35	0.10	12.66			
	*qFM-Chr5-3*	2017	Bin655	Bin656	6.06	0.10	9.81						
		2017	Bin657	Bin658				7.24	0.10	11.13			
	*qFM-Chr7-1*	2016	Bin842	Bin843				2.53	0.08	4.51			
		2016	Bin850	Bin851				2.16	0.08	3.95			
	*qFM-Chr8-1*	2018	Bin910	Bin911	2.84	0.08	5.85						
	*qFM-Chr8-2*	2018	Bin919	Bin920	2.99	0.08	6.15	2.88	0.08	6.02			
	qFM-Chr8-3*	2017	Bin964	Bin965	2.73	0.06	4.04						
		2017	Bin977	Bin978	2.02	0.06	3.01						
		2016	Bin969	Bin970				3.48	0.10	6.71			
		2016	Bin970	Bin971	2.94	0.09	5.48						
		2018	Bin977	Bin978	2.34	– 0.07	4.85	3.46	– 0.09	7.30			
		2018	Bin982	Bin983	2.84	– 0.07	5.86						
	*qFM-Chr13-1*	2018	Bin1434	Bin1435							2.49	0.05	5.41
	*qFM-Chr13-2*	2018	Bin1456	Bin1457							2.03	0.04	4.44
	*qFM-Chr13-3*	2016	Bin1473	Bin1474	2.20	0.09	3.84						
	*qFM-Chr14-1*	2017	Bin1502	Bin1503	3.11	– 0.07	4.59						
	*qFM-Chr14-2*	2016	Bin1521	Bin1522							2.54	– 0.10	5.57
		2016	Bin1528	Bin1529							2.37	– 0.09	4.99
	*qFM-Chr14-3*	2016	Bin1540	Bin1541	3.87	– 0.11	8.16						
	*qFM-Chr14-4*	2016	Bin1550	Bin1551	4.63	– 0.12	8.82						
	qFM-Chr15-1*	2016	Bin1568	Bin1569				2.08	– 0.08	4.11			
		2016	Bin1579	Bin1580				3.84	– 0.11	7.42			
		2016	Bin1580	Bin1581							3.38	0.11	7.22
		2016	Bin1589	Bin1590				2.48	– 0.09	4.88	2.42	0.10	6.45
		2018	Bin1571	Bin1572							3.55	– 0.06	7.37
		2018	Bin1594	Bin1595							4.94	0.07	10.49
	*qFM-Chr15-2*	2017	Bin1678	Bin1679				2.02	– 0.05	2.84			
	*qFM-Chr15-3*	2016	Bin1690	Bin1691				3.48	– 0.10	6.72			
		2017	Bin1690	Bin1691				2.48	– 0.06	3.84			
		2017	Bin1689	Bin1690	5.53	– 0.10	9.13						
	*qFM-Chr16-1*	2016	Bin1817	Bin1818							2.03	– 0.09	4.38
		2016	Bin1821	Bin1822				2.11	0.08	3.92	3.10	– 0.11	6.59
	*qFM-Chr19-1*	2017	Bin2061	Bin2062							3.00	– 0.05	6.15
	*qFM-Chr19-2*	2017	Bin2073	Bin2074							2.37	– 0.04	4.88
	*qFM-Chr21-1*	2017	Bin2279	Bin2280				2.05	– 0.05	2.71			
		2016	Bin2285	Bin2286	2.00	0.08	3.77						
		2016	Bin2292	Bin2293	2.77	0.09	5.15						
	*qFM-Chr21-2*	2017	Bin2330	Bin2331							2.75	0.06	5.77
	*qFM-Chr22-1*	2017	Bin2464	Bin2465	3.11	– 0.07	4.97						
	*qFM-Chr22-2*	2017	Bin2472	Bin2473				10.29	– 0.11	16.03			
		2017	Bin2473	Bin2474	2.44	– 0.06	3.94						
	*qFM-Chr23-1*	2017	Bin2537	Bin2538				2.06	0.05	2.73			
	*qFM-Chr25-1*	2016	Bin2700	Bin2701	2.48	0.09	4.91				2.06	0.08	4.32
		2016	Bin2706	Bin2707	2.47	0.08	4.56						
	*qFM-Chr25-2*	2018	Bin2726	Bin2727				2.07	– 0.07	4.42			
	*qFM-Chr25-3*	2018	Bin2738	Bin2739				3.29	– 0.09	6.92			
	*qFM-Chr25-4*	2018	Bin2751	Bin2752				2.05	– 0.07	4.39			
	*qFM-Chr26-1*	2016	Bin2810	Bin2811				2.37	– 0.08	4.52			
	*qFM-Chr26-2*	2016	Bin2837	Bin2838	4.48	– 0.12	8.74						
	*qFM-Chr26-3*	2016	Bin2845	Bin2846	5.55	– 0.13	10.69						

^a^QTL noted by ‘*’ referred to common QTL detected on two datasets at least two years

^b^Phenotypic variation explained by a single locus QTL

^c^E1, salt stress condition; E2, normal condition; *D*-value, the difference values between salt stress and normal conditions

For FL, a total of 40 QTL located on 19 chromosomes were identified. Of those, 14 QTL was detected under E1, 17 and 11 QTL were identified in RIL population under E1 and *D*-value, respectively (Table 5). Seven QTL detected on E1 increased FL with threshold of LOD 2.62 on average. Ten QTL decreased FL explained 6.53% of PV on average. The *qFL-Chr1-1, qFL-Chr5-5, qFL-Chr24-4,* which were detected on both E1 and E2, explained 3.68, 11.65, and 4.09% of PV, respectively. Three QTL were mapped with LOD threshold 2.27, 6.72, and 3.87, respectively. Two of these QTL were identified in at least two years.

Out of 26 QTL (LOD ranged from 2.04 to 4.29), 4, 13, and 9 QTL for FU was identified on E1, E2, and *D*-value, respectively. Four QTL were identified in more than two years, but no QTL were detected in more than two growing conditions in the same year.

For FS, a total of 28 QTL were resolved explaining from 3.66 to 12.90% of PV, among which seven, 12, and 13 QTL were identified on E1, E2, and *D*-value, respectively. One QTL (*qFS-Chr25-2*) by the threshold of LOD 5.79 was detected on both E1 and *D*-value. Two of three QTL located on Chr 24 explained PV more than 10%.

Nine, 15, and five QTL for FE distributed on 18 chromosomes were identified under E1, E2, and *D*-value, respectively. The stable QTL *qFE-Chr20-3*, identified in the *D*-value, was also identified on E2 in 2016 and 2017, with the threshold of LOD 3.44 on average. The QTL, *qFE-Chr7-1*, was identified under E2 and *D*-value with the threshold of LOD 4.31 on average. Identified in both E1 and *D*-value, *qFE-Chr15-1* exhibited average LOD of 2.31.

A total of 12, 18, and 8 QTL underlying FM were identified on 16 chromosomes under E1, E2, and *D*-value, respectively. Of these QTL, *qFM-Chr8-3*, *qFM-Chr15-1*, *qFM-Chr15-3*, and *qFM-Chr21-1* were detected repeatedly across two or three years. Three QTL detected on E1 also played important roles in controlling FM on E2, including *qFM-Chr5-2*, *qFM-Chr8-2*, and *qFM-Chr8-3*. Another two QTL were detected on E2 and *D*-value; these were *qFM-Chr15-1* and *qFM-Chr16-1.* One QTL, *qFM-Chr25-1*, explained 4.60% of PV and was identified under E1 and *D*-value.

### Pleiotropic effects analysis on FL and FS

A total of 35 clusters located on 18 chromosomes showed pleiotropic effects involving 107 QTL (Table 6). Of these, four clusters existed on chromosome (Chr) 5, Chr7, and Chr24; three were detected on Chr15 and Chr25; two were detected on Chr8, Chr14, Chr16, and Chr21, and only one cluster was detected on other 9 chromosomes.

**Table 6:** Pleiotropic regions in the present study for fiber quality traits

Cluster	QTL	Year	Flanking markers		Under E1^c^			Under E2			Mapping by *D*-value		
					LOD	Additive	Var%^b^	LOD	Additive	Var%	LOD	Additive	Var%
Loci-Chr2-1	*qFE-Chr2-1*	2017	Bin128	Bin129							2.95	− 0.02	6.44
	*qFM-Chr2-1*	2017	Bin128	Bin129				2.71	− 0.06	4.02			
	*qFS-Chr2-1*	2017	Bin139	Bin140							2.26	− 0.32	4.11
Loci-Chr4-1	*qFU-Chr4-1*	2017	Bin472	Bin473							3.10	− 0.26	6.48
	*qFM-Chr4-2*	2017	Bin484	Bin485				3.99	− 0.07	5.72			
Loci-Chr5-1	*qFM-Chr5-1*	2017	Bin488	Bin489				2.21	0.05	2.91			
	*qFU-Chr5-1*	2017	Bin492	Bin493				2.66	− 0.20	5.18			
	*qFE-Chr5-1*	2016	Bin493	Bin494				5.49	− 0.03	11.13			
	*qFS-Chr5-1*	2016	Bin488	Bin489	3.60	− 0.31	6.50						
	*qFS-Chr5-2*	2016	Bin500	Bin501	2.31	− 0.25	4.24						
		2016	Bin502	Bin503				2.31	− 0.25	4.04			
	*qFL-Chr5-2*	2016	Bin500	Bin501	2.79	− 0.26	4.87						
	*qFL-Chr5-1*	2016	Bin490	Bin491				4.61	− 0.34	8.20			
		2017	Bin493	Bin494	6.49	− 0.40	12.11						
	*qFL-Chr5-2*	2017	Bin502	Bin503	5.08	− 0.36	9.66						
Loci-Chr5-2	*qFL-Chr5-3*	2016	Bin512	Bin513				5.87	− 0.37	10.31			
		2017	Bin515	Bin516				6.23	− 0.36	11.06			
	*qFL-Chr5-4*	2017	Bin527	Bin528				6.90	− 0.38	12.14			
	*qFE-Chr5-2*	2016	Bin521	Bin522				3.26	0.02	6.42			
	*qFE-Chr5-3*	2016	Bin530	Bin531				2.13	0.02	4.36			
Loci-Chr5-3	*qFS-Chr5-3*	2017	Bin548	Bin549	2.22	− 0.33	3.90						
	*qFU-Chr5-2*	2016	Bin554	Bin555							2.45	0.22	4.99
Loci-Chr5-4	*qFS-Chr5-5*	2016	Bin638	Bin639				2.98	− 0.29	5.61			
	*qFS-Chr5-6*	2017	Bin640	Bin641	3.98	− 0.45	7.06						
	*qFS-Chr5-7*	2017	Bin653	Bin654	5.75	− 0.54	9.98						
		2016	Bin654	Bin655				5.75	− 0.40	10.42			
		2016	Bin655	Bin656	2.28	− 0.25	4.06						
	qFL-Chr5-5*^a^	2016	Bin648	Bin649	5.91	− 0.39	10.80	6.73	− 0.40	11.65			
		2017	Bin648	Bin649				3.29	− 0.26	5.68			
	qFM-Chr5-2*	2017	Bin641	Bin642				6.00	0.09	9.37			
		2017	Bin645	Bin646	4.85	0.09	7.98	8.35	0.10	12.66			
	*qFM-Chr5-3*	2017	Bin655	Bin656	6.06	0.10	9.81						
		2017	Bin657	Bin658				7.24	0.10	11.13			
Loci-Chr7-1	*qFS-Chr7-1*	2017	Bin789	Bin790				2.36	− 0.31	4.35			
	*qFL-Chr7-1*	2016	Bin795	Bin796							2.49	0.23	5.57
Loci-Chr7-2	*qFM-Chr7-1*	2016	Bin842	Bin843				2.53	0.08	4.51			
		2016	Bin850	Bin851				2.16	0.08	3.95			
	*qFU-Chr7-2*	2017	Bin840	Bin841				2.20	− 0.18	4.39			
		2018	Bin857	Bin858				2.24	− 0.20	4.62			
Loci-Chr7-3	*qFU-Chr7-3*	2018	Bin867	Bin868				2.22	− 0.20	4.59			
	*qFS-Chr7-3*	2018	Bin868	Bin869				2.27	− 0.41	5.11			
Loci-Chr7-4	*qFE-Chr7-1*	2018	Bin872	Bin873				3.33	− 0.01	6.80			
		2018	Bin879	Bin880							5.28	0.02	11.19
	*qFS-Chr7-4*	2017	Bin880	Bin881	2.10	− 0.33	3.74						
	*qFU-Chr7-4*	2018	Bin881	Bin882				2.05	− 0.19	4.25			
Loci-Chr8-1	*qFM-Chr8-1*	2018	Bin910	Bin911	2.84	0.08	5.85						
	*qFS-Chr8-1*	2018	Bin912	Bin913							2.27	− 0.37	4.85
Loci-Chr8-2	qFM-Chr8-3*	2017	Bin964	Bin965	2.73	0.06	4.04						
		2016	Bin969	Bin970				3.48	0.10	6.71			
		2016	Bin970	Bin971				2.94	0.09	5.48			
		2018	Bin977	Bin978				2.34	− 0.07	4.85			
		2018	Bin977	Bin978				3.46	− 0.09	7.30			
		2017	Bin977	Bin978	2.02	0.06	3.01						
		2018	Bin982	Bin983	2.84	− 0.07	5.86						
	*qFS-Chr8-2*	2017	Bin975	Bin976	3.44	− 0.42	6.14						
		2017	Bin980	Bin981	2.75	− 0.38	5.00						
Loci-Chr12-1	*qFE-Chr12-1*	2016	Bin1364	Bin1365	2.08	0.02	4.65						
	*qFL-Chr12-1*	2018	Bin1368	Bin1369							2.97	− 0.22	6.17
		2018	Bin1371	Bin1372				2.72	0.27	5.45			
Loci-Chr13-1	*qFU-Chr13-2*	2017	Bin1449	Bin1450				2.55	0.20	5.03			
	*qFU-Chr13-3*	2018	Bin1467	Bin1468							2.15	0.30	4.54
	*qFM-Chr13-2*	2018	Bin1456	Bin1457							2.03	0.04	4.44
	*qFM-Chr13-3*	2016	Bin1473	Bin1474	2.20	0.09	3.84						
Loci-Chr14-1	*qFE-Chr14-1*	2018	Bin1494	Bin1495	3.65	− 0.01	7.66						
	*qFE-Chr14-2*	2018	Bin1508	Bin1509	4.60	− 0.01	9.54						
		2018	Bin1516	Bin1517	3.17	− 0.01	6.68						
	*qFS-Chr14-1*	2016	Bin1501	Bin1502				3.08	0.30	5.42			
	*qFU-Chr14-1*	2018	Bin1520	Bin1521				3.52	0.26	8.16			
	*qFM-Chr14-1*	2017	Bin1502	Bin1503	3.11	− 0.07	4.59						
	*qFM-Chr14-2*	2016	Bin1521	Bin1522							2.54	− 0.10	5.57
		2016	Bin1528	Bin1529							2.37	− 0.09	4.99
	*qFL-Chr14-2*	2018	Bin1517	Bin1518				2.13	− 0.23	4.01			
	*qFL-Chr14-3*	2016	Bin1529	Bin1530							2.13	0.21	4.40
Loci-Chr14-2	*qFM-Chr14-4*	2016	Bin1550	Bin1551	4.63	− 0.12	8.82						
	*qFE-Chr14-3*	2018	Bin1553	Bin1554				2.25	0.01	4.60			
Loci-Chr15-1	qFM-Chr15-1*	2016	Bin1568	Bin1569				2.08	− 0.08	4.11			
		2018	Bin1571	Bin1572							3.55	− 0.06	7.37
		2016	Bin1579	Bin1580				3.84	− 0.11	7.42			
		2016	Bin1580	Bin1581				3.38	0.11	7.22			
		2016	Bin1589	Bin1590				2.48	− 0.09	4.88	2.42	0.10	6.45
		2018	Bin1594	Bin1595							4.94	0.07	10.49
	*qFL-Chr15-1*	2017	Bin1599	Bin1600							2.47	− 0.23	5.15
	*qFU-Chr15-1*	2016	Bin1579	Bin1580				2.97	− 0.18	5.70			
	*qFU-Chr15-2*	2016	Bin1599	Bin1600							2.65	0.23	5.54
	*qFE-Chr15-1*	2016	Bin1589	Bin1590	2.04	0.02	4.52						
		2016	Bin1596	Bin1597							2.80	0.02	5.68
		2016	Bin1599	Bin1600	2.08	0.02	4.22						
Loci-Chr15-2	*qFS-Chr15-1*	2017	Bin1659	Bin1660				2.14	0.29	3.73			
	*qFM-Chr15-2*	2017	Bin1678	Bin1679				2.02	− 0.05	2.84			
Loci-Chr15-3	*qFM-Chr15-3*	2017	Bin1689	Bin1690	5.53	− 0.10	9.13						
		2016	Bin1690	Bin1691				3.48	− 0.10	6.72			
		2017	Bin1690	Bin1691				2.48	− 0.06	3.84			
	*qFL-Chr15-2*	2017	Bin1694	Bin1695				2.31	0.22	3.71			
		2018	Bin1707	Bin1708				2.02	0.23	4.00			
Loci-Chr16-1	*qFE-Chr16-1*	2016	Bin1718	Bin1719				2.94	− 0.02	5.94			
	*qFL-Chr16-1*	2016	Bin1760	Bin1761				2.13	− 0.21	3.26			
Loci-Chr16-2	*qFL-Chr16-2*	2017	Bin1784	Bin1785				2.10	− 0.20	3.38			
	qFM-Chr16-1*	2016	Bin1817	Bin1818							2.03	− 0.09	4.38
		2016	Bin1821	Bin1822				2.11	0.08	3.92	3.10	− 0.11	6.59
Loci-Chr18-1	*qFU-Chr18-1*	2016	Bin1904	Bin1905	2.45	0.20	5.13						
	*qFS-Chr18-1*	2017	Bin1909	Bin1910							3.33	0.39	6.51
Loci-Chr19-1	*qFU-Chr19-1*	2017	Bin2057	Bin2058	2.28	− 0.17	4.97						
	*qFM-Chr19-1*	2017	Bin2061	Bin2062							3.00	− 0.05	6.15
	*qFS-Chr19-1*	2017	Bin2066	Bin2067							2.75	0.36	5.35
Loci-Chr21-1	*qFM-Chr21-1*	2017	Bin2279	Bin2280				2.05	− 0.05	2.71			
		2016	Bin2285	Bin2286	2.00	0.08	3.77						
		2016	Bin2292	Bin2293	2.77	0.09	5.15						
	*qFE-Chr21-1*	2018	Bin2279	Bin2280							4.21	− 0.02	8.83
Loci-Chr21-2	*qFM-Chr21-2*	2017	Bin2330	Bin2331							2.75	0.06	5.77
	*qFE-Chr21-2*	2017	Bin2336	Bin2337				2.56	0.02	4.76			
	*qFE-Chr21-3*	2017	Bin2345	Bin2346	2.28	0.02	4.37						
	*qFL-Chr21-1*	2017	Bin2335	Bin2336				2.84	0.24	4.87			
	*qFL-Chr21-2*	2016	Bin2338	Bin2339				3.19	0.28	5.65			
		2016	Bin2347	Bin2348				2.25	0.24	4.10			
Loci-Chr22-1	*qFS-Chr22-1*	2018	Bin2411	Bin2412							2.72	0.52	6.64
	*qFE-Chr22-2*	2017	Bin2412	Bin2413				2.55	0.02	4.44			
Loci-Chr23-1	*qFL-Chr23-1*	2018	Bin2499	Bin2500							3.22	0.22	6.72
		2018	Bin2503	Bin2504	2.11	0.24	4.73						
	*qFL-Chr23-2*	2018	Bin2521	Bin2522	2.83	− 0.33	5.93						
	*qFM-Chr23-1*	2017	Bin2537	Bin2538				2.06	0.05	2.73			
	*qFU-Chr23-1*	2017	Bin2554	Bin2555							2.05	0.21	4.38
Loci-Chr24-1	*qFU-Chr24-1*	2016	Bin2558	Bin2559							3.37	− 0.30	6.93
	*qFL-Chr24-1*	2017	Bin2558	Bin2559	2.67	0.26	4.74						
Loci-Chr24-2	*qFS-Chr24-1*	2017	Bin2578	Bin2579	6.25	0.56	11.19						
		2016	Bin2584	Bin2585	4.06	0.35	7.94						
		2017	Bin2579	Bin2580				2.90	0.35	5.39			
		2016	Bin2581	Bin2582				2.60	0.27	4.66			
	*qFS-Chr24-2*	2017	Bin2592	Bin2593	7.19	0.60	12.73						
		2016	Bin2593	Bin2594	6.83	0.44	12.90						
		2017	Bin2596	Bin2597				2.74	0.34	5.10			
	*qFE-Chr24-1*	2018	Bin2584	Bin2585	3.70	0.01	7.59						
	*qFU-Chr24-2*	2016	Bin2586	Bin2587							2.23	0.24	4.52
	*qFL-Chr24-2*	2016	Bin2581	Bin2582				2.66	0.25	4.52			
		2016	Bin2587	Bin2588	2.61	0.26	4.67						
		2017	Bin2587	Bin2588				3.27	0.26	5.68			
	*qFL-Chr24-3*	2016	Bin2593	Bin2594				4.08	0.31	6.80			
Loci-Chr24-3	*qFS-Chr24-3*	2017	Bin2601	Bin2602	4.78	0.51	8.72						
	qFL-Chr24-4*	2016	Bin2606	Bin2607	2.27	0.25	4.09	2.13	0.23	3.64			
Loci-Chr24-4	*qFL-Chr24-5*	2017	Bin2627	Bin2628							2.10	0.22	4.47
	*qFU-Chr24-3*	2017	Bin2630	Bin2631	2.74	0.19	5.78						
Loci-Chr25-1	*qFL-Chr25-1*	2017	Bin2674	Bin2675							2.07	− 0.21	4.35
	*qFU-Chr25-1*	2016	Bin2681	Bin2682	2.20	0.18	4.54						
	*qFL-Chr25-2*	2017	Bin2683	Bin2684							4.62	− 0.31	10.09
Loci-Chr25-2	*qFE-Chr25-1*	2017	Bin2695	Bin2696							2.90	− 0.02	5.95
	qFM-Chr25-1*	2016	Bin2700	Bin2701	2.48	0.09	4.91				2.06	0.08	4.32
		2016	Bin2706	Bin2707	2.47	0.08	4.56						
	*qFS-Chr25-1*	2016	Bin2707	Bin2708	2.03	− 0.24	3.66						
		2016	Bin2712	Bin2713	2.67	− 0.28	4.78						
Loci-Chr25-3	qFS-Chr25-2*	2016	Bin2769	Bin2770	3.21	0.30	5.79				2.17	0.27	4.95
	*qFU-Chr25-2*	2018	Bin2763	Bin2764							2.70	− 0.31	5.72
		2018	Bin2774	Bin2775							2.76	− 0.32	5.85
Loci-Chr26-1	*qFL-Chr26-1*	2016	Bin2808	Bin2809	2.09	0.23	3.65						
	*qFM-Chr26-1*	2016	Bin2810	Bin2811				2.37	− 0.08	4.52			

^a^QTL noted by ‘*’ referred to common QTL detected on two datasets at least two years

^b^Phenotypic variation explained by a single locus QTL

^c^E1, salt stress condition; E2, normal condition; *D*-value, the difference values between salt stress and normal conditions

There are two pleiotropic regions that can improve five fiber quality traits, which are Loci-Chr5-1 and Loci-Chr14-1, respectively. Two clusters could improve four fiber quality traits, which including Loci-Chr15-1 (FL, FU, FE FM), Loci-Chr24-2 (FL, FU, FE, FS). Six clusters controlled three traits, which were Loci-Chr5-4 (FL, FS, FM), Loci-Chr7-4 (FU FS, FE), Loci-Chr19-1 (FU, FS, FM), Loci-Chr21-2 (FL, FE, FM), Loci-Chr23-1 (FL, FE, FM), and Loci-Chr25-2 (FS, FE, FM), respectively.

Among the 35 pleiotropic regions, six affected FL and FS simultaneously, which were distributed on Chr5, Chr7, Chr14, and Chr24, of which two regions were found in both Chr5 and Chr24. Loci-Chr5-1, Loci-Chr5-4, and Loci-Chr7-1, controlled FL and FS simultaneously and received favorable alleles contributed by the female GX1135. Among them, the two QTL *qFS-Chr5-1* and *qFS-Chr5-2* that controlled FS on Loci-Chr5-1 were detected in E1 and E2 in 2016, and the two QTL *qFL-Chr5-1* and *qFL-Chr5-2* that control FL were detected in E1 and E2 in 2016 and 2017 simultaneously. The cluster also controlled FU, FE, and FM. The Loci-Chr5-4 cluster contains six QTL, of which three QTL controlled FS (*qFS-Chr5-5, qFS-Chr5-6* and *qFS-Chr5-7*), one controlled FL (*qFL-Chr5-5*), which detected in E1 and E2 in 2016 and 2017, and the region also controls FM (*qFM-Chr5-2, qFM-Chr5-3*). Two QTL were detected on Loci-Chr7-1, of which, *qFS-Chr7-1*, affecting FS was detected in E2 in 2017, but *qFL-Chr7-1* was detected in E3 in 2016. Loci-Chr24-2 and Loci-Chr24-3, two pleiotropic regions on chromosome 24, were detected in both E1 and E2 in 2016 and 2017, and the favorable alleles were contributed by male parent GX100-2. Among them, two QTL influencing FL and FS in Loci-Chr24-2, (*qFS-Chr24-1*, *qFS-Chr24-2* and *qFL-Chr24-2*, *qFL-Chr24-3*), which also control FE and FU simultaneously. Eight QTL were detected in Loci-Chr14-1, of which only one QTL (*qFS-Chr14-1*) influencing FS was detected in E2, 2016, two QTL from Loci-Chr14-1 influencing FL were detected in E2, 2018. The favorable alleles in *qFS-Chr14-1* and *qFL-Chr14-2* originated from the female GX1135, while the favorable alleles in *qFL-Chr14-3* were contributed by male GX100-2.

### Identification of candidate genes within QTL and expression validation by RNA-seq

**Fig. 1:**
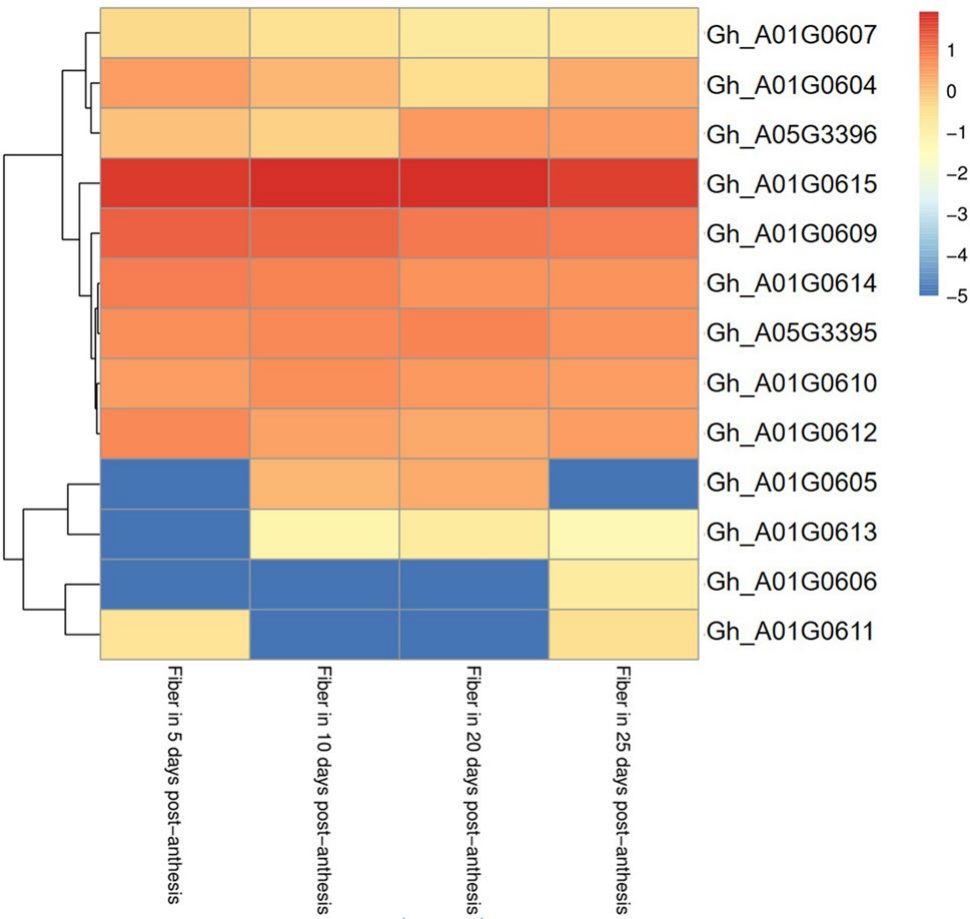
Expression profiles of candidate genes in fiber development identified within important QTL

In this study, two QTL (*qFL-Chr1-1* and *qFL-Chr5-5*) influencing FL were detected in both E1 and E2 and detected repeatedly across two years. Explaining 4.26% of the average PV, *qFL-Chr1-1*, is favorably provided by the female parent. The QTL, *qFL-Chr5-5*, is provided by male parent, and the average PV is 9.38%. Among them, the confidence interval of *qFL-Chr1-1* is between marker Bin28 and Bin29, corresponding to the reference genome of upland cotton TM-1 10,876,918 bp to 11,016,009 bp, the physical distance of this interval is 139,091 bp, which includes 12 annotated genes (*Gh_A01G0604-Gh_A01G0615*). The flanking markers of *qFL-Chr5-5* are Bin648 and Bin649, corresponding to the reference genome of upland cotton 88,623,793 bp to 88,671,394 bp, the physical distance of this region is 47,601 bp, including two annotated genes (*Gh_A05G3395* and *Gh_A05G3396*) (Table 7). Among them, there are 11 known genes and three genes were with uncharacterized protein information. The known genes were annotated as Microtubule-associated protein TORTIFOLIA1, DNA polymerase I A, UDP-glycosyltransferase 73C5, Serine/threonine-protein kinase PBS1, Vacuolar protein sorting-associated protein, Fatty acid desaturase 4, Cytochrome P450 716B1, Protein FATTY ACID EXPORT 5, Polyadenylate-binding protein RBP45C, Probable protein phosphatase 2C 33, and Aspartic proteinase-like protein 2, respectively. These putative candidate genes were explored for the public RNA-seq expression data of TM-1 in fiber development (Fig. 1). The result showed that fragments per kilobase million (FPKM) of four genes (*Gh_A01G0606, Gh_A01G0607, Gh_A01G0611, and Gh_A01G0613*) were less than 1. The gene (*Gh_A01G0615*) is persistently highly expressed at different stages of fiber development. Three genes (*Gh_A01G0604, Gh_A01G0605, and Gh_A01G3396*) were highly expressed at various stages of fiber development (5, 10, and 20 DPA).

**Table 7:** Putative candidate genes identified within important QTL genomic regions

QTL	Gene ID	Gene name	Description
*qFL-Chr01-1*	Gh_A01G0604	TOR1	Microtubule-associated protein TORTIFOLIA1
	Gh_A01G0605	POLIA	DNA polymerase I A, chloroplastic/mitochondrial
	Gh_A01G0606	UGT73C5	UDP-glycosyltransferase 73C5
	Gh_A01G0607	PBS1	Serine/threonine-protein kinase PBS1
	Gh_A01G0608	NA	NA
	Gh_A01G0609	NA	NA
	Gh_A01G0610	VP22-1	Vacuolar protein sorting-associated protein 22 homolog 1
	Gh_A01G0611	FAD4	Fatty acid desaturase 4, chloroplastic
	Gh_A01G0612	NA	NA
	Gh_A01G0613	CYP716B1	Cytochrome P450 716B1
	Gh_A01G0614	FAX5	Protein FATTY ACID EXPORT 5
	Gh_A01G0615	RBP45C	Polyadenylate-binding protein RBP45C
*qFL-Chr05-5*	Gh_A05G3395	PPC6-1	Probable protein phosphatase 2C 33
	Gh_A05G3396	At1g65240	Aspartic proteinase-like protein 2

**Fig. 2:**
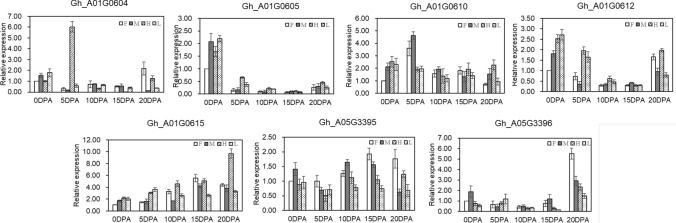
The relative expression levels of seven candidate genes identified in extreme lines and parents using qRT-PCR. The genes relative expression levels were determined by 2^−ΔΔCT^ as expressed and were normalized to the expression level of *GhUBQ7* gene. F, GX1135; M, GX100-2; H, extremely long fiber length line; L, extremely short fiber length line

To validate the potential function in fiber development, the expression pattern of candidate genes was verified with qRT-PCR using fibers of extremely long fiber line (H), extremely short fiber line (L), female (GX1135, F), male (GX100-2, M) at different developmental stages (Fig. 2). The expression of *Gh_A01G0604* in extremely long fiber line was six times higher than that in extremely short fiber line at 5 DPA. The expression of *Gh_A01G0610, Gh_A01G0612, Gh_A01G0615, Gh_A01G3395*, and *Gh_A01G3396* in extremely long fiber line was significantly higher than that of extremely short fiber line at many points from 10 to 20 DPA. Therefore, these seven genes were identified as candidate genes responsible for influencing fiber cell elongation.

### Genes within QTL for FL conferring fatty acid synthesis and degradation

Among the 40 QTL identified for FL, 9 were detectable in *D*-value, 13 positive and 18 negative effect QTL among the remaining 31 QTL were observed on E1 or E2, respectively. This examination failed to find the corresponding physical location of some SSR markers on the genome, 10 of which were QTL with positive effect and 11 QTL with negative effect for FL were subjected to gene functional prediction. The physical locations of these QTL on the reference genome of *G. hirsutum* were found, and the numbers of genes in the corresponding regions were 977 and 791, respectively (Table S7).

We annotated these genes by GO and classified the annotated GO terms. Among them, genes in the QTL region of positive and negative effects were divided into 52 subgroups, belonging to three major categories, CC (cellular components), MF (molecular function), and BP (biological processes) (Fig. S2). There was no significant difference between genes with positive and negative effect QTL in GO classification. GO enrichment of these two kinds of genes showed that the positive effect genes were mainly enriched in three GO items: transmembrane receptor protein tyrosine kinase activity in BP, cell periphery in CC, and system development in MF. The negative effect genes were mainly enriched in abscisic acid glucosyltransferase of BP, membrane-bounded organelle in CC and cellular protein modification processes in MF (Fig. S3).

**Fig. 3:**
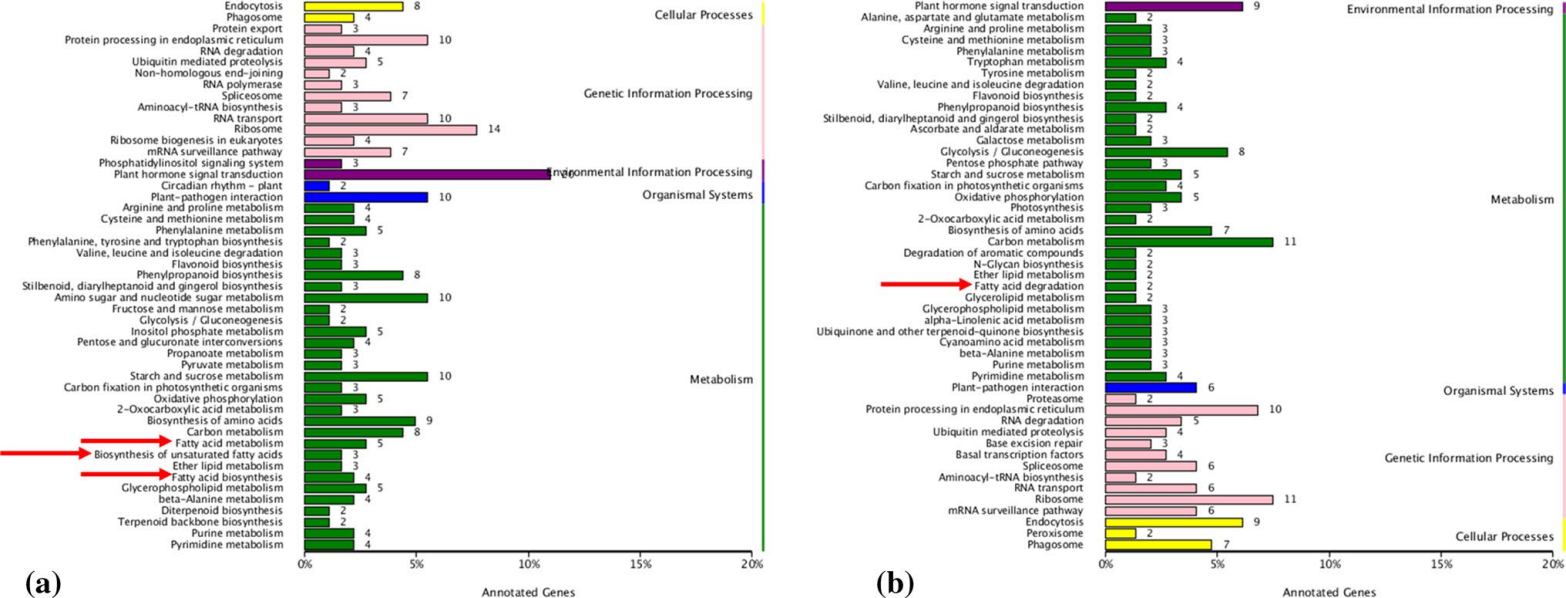
KEGG functional annotation and classification of genes in positive and negative effect QTL controlling fiber length. **a** KEGG functional annotation and classification of genes within the positive effect QTL. **b** KEGG functional annotation and classification of genes within the negative effect QTL

**Fig. 4:**
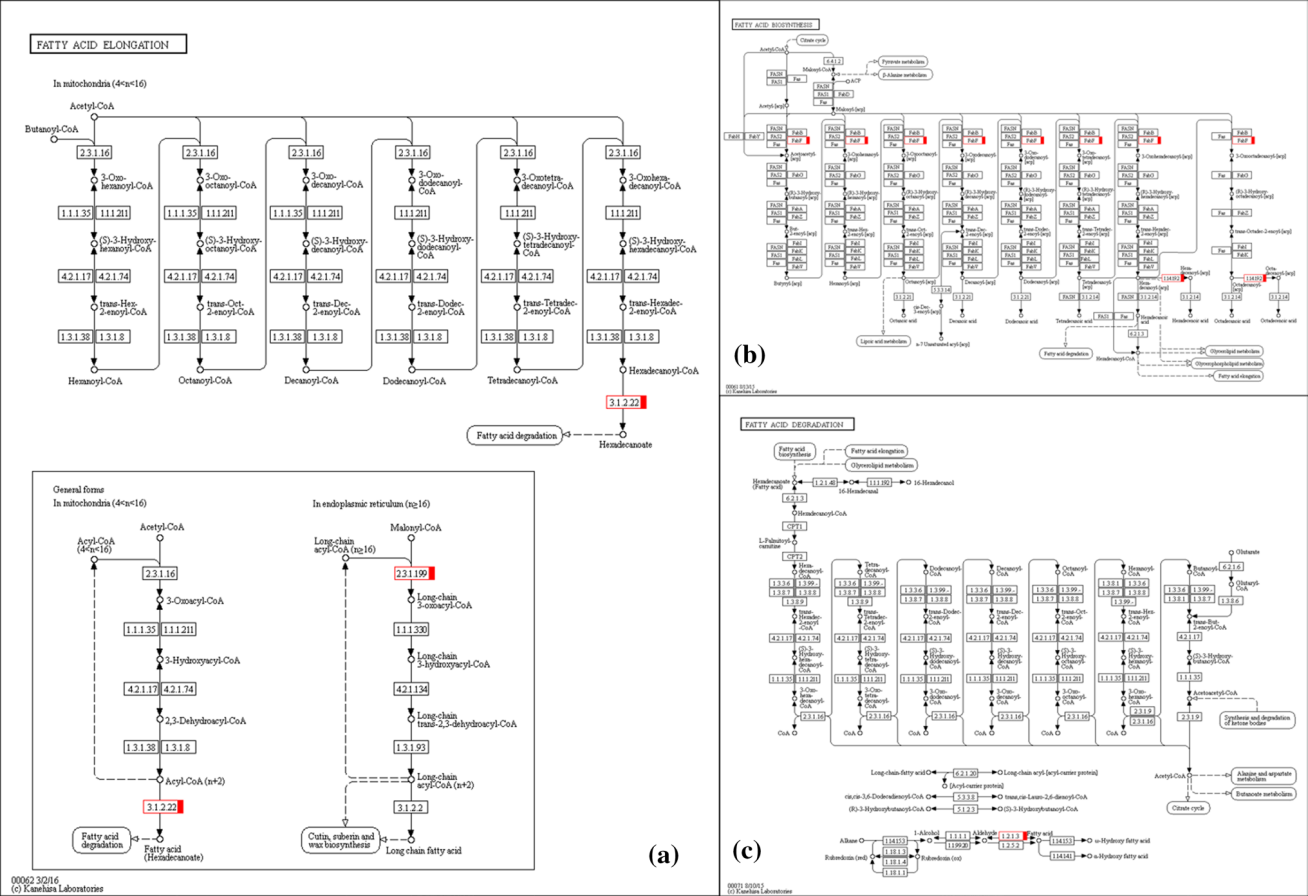
Genes involved in fatty acid metabolism. **a** Genes involved in fatty acid elongation pathway. **b** Genes involved in fatty acid biosynthetic pathway. **c** Genes involved in fatty acid degradation pathway

To better understand the biological function of candidate genes and their metabolic pathways, all genes (70,478) in upland cotton (Zhang et al. 2015) serve as background and the candidate genes in positive and negative effect QTL were annotated and enriched by KEGG (Fig. 3 and Fig. S4). KEGG annotation showed that the genes in positive QTL were mainly concentrated in three metabolic pathways: plant hormone signal transduction, carbon metabolism and ribosomal, while the genes in negative effect QTL were mainly enriched in plant hormone signal transduction, carbon metabolism, plant pathogen interaction, ribosomes and endocytosis. Interestingly, we found that genes with positive and negative effect QTL are involved in fatty acid metabolism pathways, in which positive effect genes participate in ko00061 (Fatty acid biosynthesis), ko00062 (Fatty acid elongation) and ko01040 (Biosynthesis of unsaturated fatty acids); negative effect genes participate in ko00071 (Fatty acid degradation) (Fig. 4). There are two genes (*Gh_D08G1373* and *Gh_D08G1533*) involved in fatty acid elongation pathway six genes involved in fatty acid biosynthesis pathway and two genes involved in fatty acid degradation pathway (Table S8).

## Discussion

### The salinity effects on cotton growth and fiber quality traits

According to the classification standard of soil salt content, in the field trails from 2016 to 2018, the classification of soil salinity is moderate salinization in salt stress condition, and mild salinization in normal condition, as well as the depth of 0–20 cm (Table S1, S2). It can be preliminarily considered that salt treatment is consistent and effective.

By observing the cotton performance in the field, we found that compared with the cotton planted in control soil, the plant height of cotton grown in saline soil was shorter, and leaf margins curled and wilted along the leaf vein. We saw that salt stress negatively affected the vegetative growth of cotton (Fig. S2). The tendency of fiber quality to change among lines in the RIL population under E1 and E2 was different from 2016 to 2018. This could be due to the increase in soil salt content in 2018 and the uneven distribution of salt in the experimental area. It has been reported that low salinity can promote plant growth, but little research has been published to expound the molecular mechanisms of the effects of salt stress on fiber quality in cotton. Salt stress has been shown to result in a considerable decrease in the fresh and dry weights of roots, stems, and leaves (Chartzoulakis and Klapaki 2000; Su et al. 2020). It is reported that in *Alhagi pseudoalhagi* (a leguminous plant), plant weight increased at low salinity (50 mM NaCl) but decreased at high salinity (100 and 200 mM NaCl) (Kurban H. et al. 1999).

### QTL conferring fiber quality under salt stress

The identification of QTL across multiple environments and populations plays an essential role in marker-assisted selection (MAS) (Jamshed et al. 2016). A total of 318 QTL for FL in upland cotton have been submitted in CottonQTLdb database (https://www.cottonqtldb.org:8081). In the present study, 12 QTL were detected over two years, of which seven, one, four, one and one QTL were detected for FL, FU, FS, FE, and FM. The QTL detected in at least two datasets during the same year were defined as stable QTL. All of 10 stable QTL showed effect values by alleles with different parents. There are two stable QTL underlying FL (*qFL-Chr1-1* and *qFL-Chr24-4)*, one (*qFS-Chr25-2*) controlling FS and three (*qFM-Chr5-2*, *qFM-Chr8-2* and *qFM-Chr25-1*) for FM were provided positive provided alleles by the female parent. A total of six QTL were detected in both E1 and E2. Three QTL controlling FL were detected in 2016, of which, *qFL-Chr1-1* and *qFL-Chr24-4* were provided alleles by the female parent and *qFL-Chr5-5* was provided alleles positive by the male parent. Two of the QTL that affect FM are located on Chr8 (*qFM-Chr8-2*, *qFM-Chr8-3*) and were detected in 2018. One stable QTL (*qFM-Chr5-2*) was detected in 2017, where *qFM-Chr5-2* and *qFM-Chr8-2* demonstrated positive additive effects, and the other QTL, *qFM-Chr8-3* showed negative additive effects. The results revealed that these hotspots play important roles in two conditions for diverse fiber quality traits but different mechanisms responded between under E1 and E2.

Additionally, two QTL (*qFM-Chr25-1*, *qFS-Chr25-2)* were detected in E1 and *D*-value in 2016. The favorable alleles were provided by the female parent for both FM and FS. Two QTL were detected in E2 and *D*-value in 2016, affecting FM, *qFM-Chr15-1*, the positive allele of which was provided by the male parent and *qFM-Chr16-1*, the positive allele of which was provided by the female parent. A total of 46 QTL were detected for abiotic and biotic stress resistance in cotton on Chr5 including three hotspots, and 22 QTL on Chr14 including 1 hotspot, and 31 QTL on Chr15 including 2 hotspots (Abdelraheem et al. 2017). The hotspot named c15-ST-Hotspot-1 was detected for fresh root weight under salt stress condition. The stable QTL, *qFE-Chr25-1* in the present study, was detected in *D*-value, consistently with the same chromosome harboring a hotspot for Micronaire under normal irrigation condition (Said et al. 2013) and two hotspots for abiotic and biotic stress resistance underlying Micronaire and fiber elongation in cotton (Abdelraheem et al. 2017). This indicates regions on the same chromosome underlying fiber quality traits showed response to diverse stress conditions. One consistent QTL located on Chr9 and four consistent QTL on Chr15 were detected for shoot height, shoot fresh weight, shoot dry weight, and root dry weight under salt stress in seedling stage (Oluoch et al. 2016).

### Stable QTL affecting FL under multiple environments across multiple years

A QTL cluster is defined as a densely populated QTL region on a chromosome that contains many QTL associated with different traits (Rong et al. 2007). In this study, we identified 35 clusters located on 18 chromosomes. Two stable QTL were located in the cluster, Loci-Chr5-4 controlling FL (*qFL-Chr5-5*) and FM (*qFM-Chr5-2*) under E1 and E2. This cluster harbored six QTL, of which three QTL controlled FS *(qFS-Chr5-5*, *qFS-Chr5-6*, *qFS-Chr5-7*) and one for FL (*qFL-Chr5-5*) had negative additive effects, while two QTL *qFM-Chr5-2* and *qFM-Chr5-3* controlled FM showed positive additive effects. Three pleiotropic regions contained two stable QTL (*qFM-Chr25-1* and *qFS-Chr25-2*) were detected on Chr25, of which it also controlled FL and FU simultaneously. This is consistent with previous research that an important cluster with more than three traits, with high broad sense heritability and high percentage of phenotypic variation was identified on Chr25 (Diouf et al. 2018). There were four QTL-clusters on Chr24, six QTL in Loci-Chr24-2, and two QTL in Loci-Chr24-1, Loci-Chr24-3, Loci-Chr24-4, respectively. In Loci-Chr24-2, six QTL had positive additive effects which controlled FL, FS, FE, and FU simultaneously. One stable QTL, *qFL-Chr24-4*, had positive additive effects that were identified in Loci-Chr24-3 on both E1 and E2, which controlled FS. Naoumkina et al. (2019) found 12 genes possessing non-synonymous SNPs (nsSNPs) significantly associated with fiber length on Chr. D11 (Chr24 in present research) using 550 RILs derived from eleven different cultivars. The high correlation of the QTL detected in this study to the previous finding, provides the opportunity for the utilization of these QTL in MAS to improve the fiber quality of upland cotton.

There were six QTL-clusters influencing FL and FS, of which two clusters (Loci-Chr5-1, Loci-Chr5-4) located on Chr5, two (Loci-Chr24-2, Loci-Chr24-1) located on Chr24, one (Loci-Chr7-1) located on Chr7, and one (Loci-Chr14-1) located on Chr14, respectively. Six clusters affecting FL and FS simultaneously received favorable alleles contributed by the same parent under E1 and E2 except Loci-Chr14-1 (Table 6). The cluster located on Chr14 indicated that the correlation between FS and FL was not altered by salt stress.

### Advantage of multiple datasets for QTL mapping under salt stress

In general, when mapping with composite traits, the QTL detected by both constituent and composite traits have a positive correlation in detecting efficacy. Constituent traits can accumulate the genetic effect; however, the efficiency of composite traits is generally high with multiplier effects (Hua et al. 2002). In recent years, composite traits in stress response have been studied using QTL mapping strategies. In a maize example, anthesis silking interval (ASI) index was used to identify the phenotypic value for drought tolerance (Ribaut et al. 1996; Messmer et al. 2009). ASI in tropical open-pollinated varieties was negatively correlated with yields under drought stress, so ASI was one of the most important composite traits in the identified drought tolerance. The phenotypic values of ASI came from the difference between MFLW (male flowering) and FFLW (female flowering). Ribaut et al. (1996) used 142 molecular markers to analyze the characters of MFLW, FFLW, and ASI of a maize F_2_ population under good irrigation and water stress conditions, respectively. In the present study, the *D*-value was used to represent the effect of salt stress on the RIL population, which was developed as a compound trait. The results showed that 46 QTL were detected in *D*-value, of which 11 could be detected in the constituent traits explaining from 4.32 to 11.19% of PV and LOD values ranged 2.06–5.28. Of the 11 QTL, four QTL (*qFS-Chr25-2, qFM-Chr15-1, qFM-Chr16-1, qFM-Chr25-1*) were detected using constituent trait and composite trait in the same year (E1 and *D*-value, E2 and *D*-value), which explained PV, ranging from 4.32% to 6.59% (Table 5). Among them, two QTL (*qFM-Chr15-1*, *qFM-Chr16-1*) were detected on E2 and *D*-value, and two (*qFM-Chr25-1*, *qFS-Chr25-2*) were detected on E1 and *D*-value. This research exploits and provides an example for QTL effects detected in both the composite trait and the constituent traits for salt-tolerant QTL mapping.

### Candidate genes positively regulate fiber elongation

Fiber length is one of the most important and highly heritable fiber quality trait, which has been successfully used for genetic analysis in cotton and is directly related to its spinning quality. Fiber development is a complex physiological and biochemical process, which goes through multiple stages and involves a multi-level and multi-pathway molecular regulation network. Among them, plant hormones, turgor regulation, and cytoskeleton can all participate in the regulation of cotton fiber growth and development (Ascencio-Ibáñez et al. 2008; Gardiner et al. 2011). Two genes, *Gh_A05G3395* and *Gh_A05G3396*, located within *qFL-Chr5-5* had homologous genes in *Arabidopsis thaliana*. *Gh_A05G3396* was a membrane-anchored aspartic protease, which contributed to pollen and ovule development. And *Gh_A05G3395* is an Mn^2+^ or Mg^2+^-dependent protein serine/threonine phosphatase, which may constitute positive regulators in ABA-mediated signaling pathways (Vanholme et al. 2014; Xue et al. 2008). Li et al. (2002) cloned a gene (*GhTUB1*), which encoded *β* subunit of micro-tubulin and predominantly expressed in cotton fiber. He et al. (2008) identified 795 tubulin ESTs (expressed sequence tags), in upland cotton, of which 19 β-TUB genes were cloned. The microtubule-associated protein encoding gene (*Gh_A01G0604*) was significant highly expressed in extremely long fiber length line at the earliest time of fiber development (5 DPA). *Gh_A01G0605* (DNA polymerase I A), *Gh_A01G0610* (vacuolar protein sorting-associated protein), *Gh_A01G0612* (no functional annotation), *Gh_A01G0615* (Polyadenylate-binding protein) in extremely long fiber line was significantly higher than that of extremely short fiber line at 10 DPA to 20 DPA. These candidate genes annotated as being associated with plant turgor regulation, cytoskeleton processes, DNA replication, and protein synthesis might positively regulate fiber elongation in *A. thaliana* or in *G. hirsutum*, and these two QTL may contribute to fiber quality breeding.

### Fiber cell elongation aroused fatty acid synthesis pathway under salt stress

In total, 977 genes in 10 positive effect QTL and 791 genes in 10 negative effect QTL were used to analyze gene function and the biological processes (Table S7). Interestingly, we found that genes with positive effects participate in ko00061 (Fatty acid biosynthesis), ko00062 (Fatty acid elongation), and ko01040 (Biosynthesis of unsaturated fatty acids); while negative effect genes involved in ko00071 (Fatty acid degradation). Of these, a gene encoding 3-ketoacyl-CoA synthase 1 belonging to *GhKCS1* (*Gh_D08G1373*) was involved in fatty acid elongation pathway (Fig. 3 and 4, Table S8). The biosynthesis of VLCFAs and their transport are required for fiber development (Hu et al. 2007). The heterologous gene *KCS* of cotton expressed in *Arabidopsis thaliana* had promoted the elongation of stem cells (Qin et al. 2007; Shi et al. 2006). Furthermore, increased biosynthesis of fatty acid during the fiber cell elongation period implicated that fatty acid was serve as precursors of cutin and cuticular wax in the process (Hu et al. 2019a, b; Fig. 4). We predict that QTL with positive effects on fiber length may increase fiber length by promoting the synthesis and elongation of fatty acids, while QTL with negative effects may be responsible for fatty acid degradation and thus inhibit elongation of fiber cells. Our research provides a foundation for future examination of the role of fatty acid in cotton fiber development under salt stress. However, the regulative mechanism of these genes in fiber elongation still needs to be further verified.

## Electronic supplementary material

Below is the link to the electronic supplementary material.

Supplementary file1 (rar 2957 kb)

## Data Availability

All data generated or analyzed in this study included in published article and additional files. All of our raw data are available as Supporting Information: Supplementary tables S3–S5.
